# On the impact of relatedness on SNP association analysis

**DOI:** 10.1186/s12863-017-0571-x

**Published:** 2017-12-06

**Authors:** Arnd Gross, Anke Tönjes, Markus Scholz

**Affiliations:** 1 Institute for Medical Informatics, Statistics and Epidemiology, University of Leipzig, Haertelstrasse 16-18, Leipzig, 04107 Germany; 2 LIFE - Leipzig Research Center for Civilization Diseases, University of Leipzig, Philipp-Rosenthal-Strasse 27, Leipzig, 04103 Germany; 3 Department of Medicine, University of Leipzig, Liebigstrasse 18, Leipzig, 04103 Germany

**Keywords:** Heritability, Linear regression, Relatedness, SNP association analysis

## Abstract

**Background:**

When testing for SNP (single nucleotide polymorphism) associations in related individuals, observations are not independent. Simple linear regression assuming independent normally distributed residuals results in an increased type I error and the power of the test is also affected in a more complicate manner. Inflation of type I error is often successfully corrected by genomic control. However, this reduces the power of the test when relatedness is of concern. In the present paper, we derive explicit formulae to investigate how heritability and strength of relatedness contribute to variance inflation of the effect estimate of the linear model. Further, we study the consequences of variance inflation on hypothesis testing and compare the results with those of genomic control correction. We apply the developed theory to the publicly available HapMap trio data (*N*=129), the Sorbs (a self-contained population with *N*=977 characterised by a cryptic relatedness structure) and synthetic family studies with different sample sizes (ranging from *N*=129 to *N*=999) and different degrees of relatedness.

**Results:**

We derive explicit and easily to apply approximation formulae to estimate the impact of relatedness on the variance of the effect estimate of the linear regression model. Variance inflation increases with increasing heritability. Relatedness structure also impacts the degree of variance inflation as shown for example family structures. Variance inflation is smallest for HapMap trios, followed by a synthetic family study corresponding to the trio data but with larger sample size than HapMap. Next strongest inflation is observed for the Sorbs, and finally, for a synthetic family study with a more extreme relatedness structure but with similar sample size as the Sorbs. Type I error increases rapidly with increasing inflation. However, for smaller significance levels, power increases with increasing inflation while the opposite holds for larger significance levels. When genomic control is applied, type I error is preserved while power decreases rapidly with increasing variance inflation.

**Conclusions:**

Stronger relatedness as well as higher heritability result in increased variance of the effect estimate of simple linear regression analysis. While type I error rates are generally inflated, the behaviour of power is more complex since power can be increased or reduced in dependence on relatedness and the heritability of the phenotype. Genomic control cannot be recommended to deal with inflation due to relatedness. Although it preserves type I error, the loss in power can be considerable. We provide a simple formula for estimating variance inflation given the relatedness structure and the heritability of a trait of interest. As a rule of thumb, variance inflation below 1.05 does not require correction and simple linear regression analysis is still appropriate.

**Electronic supplementary material:**

The online version of this article (doi:10.1186/s12863-017-0571-x) contains supplementary material, which is available to authorized users.

## Background

When testing for SNP associations in related individuals, one has to account for the non-independence of observations [1]. An appropriate method is to test for the SNP effect assuming a mixed model **y**=*b*_1_+*b*_2_ **s**+**g**+**e** with phenotypes **y**, intercept *b*_1_, effect *b*_2_, SNP genotypes **s**, polygenic random effects **g** and residuals **e** [2–5]. Recently, several extensions of this concept were proposed [6]. However, fitting this mixed model is mathematically challenging as well as computationally expensive when performed within a genome-wide context and for large sample sizes. For this reason, the correlation of phenotypes is often neglected and the standard linear model **y**=*β*_1_+*β*_2_ **s**+***ε*** is used assuming independent normally distributed residuals ***ε***.

The impact of relatedness on the correctness of simple linear regression analysis also depends on the heritability of the trait of interest. This is obvious if considering traits of high heritability such as height (80%) [7]. However, we demonstrate in the present paper that even if heritability is relatively small (e.g. circulating serum chemerin with estimated 16% heritability [8]) proper correction is still required if highly related samples are analysed. Otherwise, the type I error of the uncorrected test statistic is inflated [9, 10] and increases further with higher heritability and stronger relatedness [1, 10, 11]. In this context, stronger relatedness means more and stronger related pairs of individuals in the analysis sample. Often, inflation of type I error is corrected by genomic control, a phenomenological approach proposed by Devlin & Roeder [12]. They showed that dependency structures of observations can lead to extra variance compared to the situation of independence. Although genomic control works fine to reduce type I error inflation, it reduces the power in case of higher relatedness and heritability [5]. Assessing the power of the uncorrected test in dependence on the degree of relatedness is difficult. We showed in a simulation study [13] that for the uncorrected test under relatedness, there is a gain in power for low *p*-value thresholds but a loss in power for higher *p*-value thresholds. Another simulation study [11] reported that the power did not notably differ if relatedness is ignored.

In the present paper, we aim to investigate how heritability and strength of relatedness contribute to variance inflation of the effect estimate and present simple approximation formulae. We evaluate subsequently the impact of variance inflation on type I error and power of the test and identify situations in which simple linear regression is still valid. Additionally, we prove that the expectation of effect estimates is not influenced as noticed by simulation studies [1, 11] and explain why allele frequencies appear to have only little impact on type I error and power (see [1, 14]).

The paper is organized as follows: In the “Methods” section, we present the underlying theory and derive the equations. We first introduce the notation of relatedness structure. Then, we present both, the general linear model of SNP-phenotype association under relatedness and its counter-part of ignored relatedness. We show unbiasedness of the effect estimate of the SNP of the second model and derive its variance inflation under relatedness. We study the impact of variance inflation on hypothesis testing and compare our results with those of genomic control correction. In the “Results” section, we analyse the relatedness structure of the publicly available HapMap data, an isolated population and synthetic family structures and their impact using the derived formulae. Major formulae derived in the paper were implemented in an R script provided as Additional file 1.

## Methods

Almost all of the equations presented in the sections below are derived in Additional file 2. Notations and a list of symbols are provided in Additional file 2: Sections 1 and 7, respectively.

### Relatedness

When dealing with relatedness, it is important to understand what exactly it means that one individual “is related” to another individual. We introduce the corresponding notation following Wang [15]. We assume bi-allelic markers (SNPs) without missing genotypes throughout. SNP genotype *s*_*i*_ of the *i*th individual corresponds to the number of reference alleles 0, 1 or 2.

We denote *ϕ* and *δ* as the probabilities that only one allele and both alleles, respectively, are inherited IBD (identical by descent) from a common ancestor. Then, relatedness is defined as *G*=*ϕ*/2+*δ*. It holds that 0≤*G*≤1. Of note, different kinds of relatedness, e.g. a parent child pair (*ϕ*=1, *δ*=0) or full siblings (*ϕ*=1/2, *δ*=1/4), can yield the same *G*. In these cases the expectation of *G* equals 1/2. The true underlying relatedness structure is often unknown. However, it can be estimated on a sufficiently rich data basis such as genome-wide SNP arrays. For estimation, we applied the method described in [15]. Our analysis is based on these relatedness estimates rather than relationships obtained from pedigrees which are often not available or prone to errors. For estimation of relatedness, SNP weights are required which depend on the respective allele frequencies. For this purpose, allele frequencies for each SNP **s** were assessed by the simple estimate $\hat p=\sum ^{n}_{i=1}{s_{i}/2/n}$ for *n* samples.

For most of the approximation formulae presented below, we require that the mean relatedness, i.e. the average of the entries *G*_*ij*_, *i*≠*j*, is small, i.e. less than 0.01. This applies for example for a sufficiently large number of trios or families or even large pedigrees over several generations (see Table 1 below).

**Table 1:** Estimated variance inflation under relatedness

Study	*n*	$\bar \lambda $	$\bar \lambda _{10\%}$	*λ* ^′^	*λ* *f*;*m*;*c*′	$\bar {G}$	$R^{2}_{\mathrm {t}}$
HapMap	129	1.288 (0.074)	1.295 (0.051)	1.297	-	0.006	0.152
SFS1	129	1.284 (0.087)	1.293 (0.051)	1.294	1.295	0.007	0.153
SFS2	999	1.306 (0.050)	1.313 (0.020)	1.314	1.299	0.001	0.143
Sorbs	977	1.410 (0.135)	1.448 (0.071)	1.449	-	0.001	0.100
SFS3	999	2.006 (0.139)	2.022 (0.083)	2.021	2.002	0.002	0.044

Variance inflation and related measures are compared between the data sets HapMap, SFS1 (synthetic family study 1), SFS2, Sorbs and SFS3 assuming $R^{2}_{\mathrm {h}}=0.9$. Provided are the sample size *n*, average inflation $\bar \lambda $ of all SNPs, average inflation $\bar \lambda _{10\%}$ estimated for SNPs with minor allele frequencies > 10*%*, expected (theoretical) inflation *λ* ^′^ obtained from estimated relationships, expected inflation *λ* *f*;*m*;*c*′ obtained from true relationships (synthetic family studies only), mean relatedness $\bar {G}$ and heritability $R^{2}_{\mathrm {t}}$corresponding to inflation *λ*t′=1.05. Standard deviations are given in parentheses

### Modelling a SNP - phenotype association

We assume that phenotypes **y** follows the “true” mixed model

1 $$ y_{i}=b_{1}+b_{2} s_{i}+g_{i}+e_{i}  $$

with intercept *b*_1_, SNP effect *b*_2_, random (polygenic) effects **g**=(*g*_1_,*g*_2_,…,*g*_*n*_) and residuals **e**=(*e*_1_,*e*_2_,…,*e*_*n*_) for *i*=1,2,…,*n* observations. For the random effects, we assume that $\mathbf {g}\sim \mathrm {N}_{n}\left (0,\sigma ^{2}_{\mathrm {g}}\mathbf {G}\right)$ is multivariate normal with a certain variance $\sigma ^{2}_{\mathrm {g}}$ and relatedness matrix **G**. The possible dependence of phenotypes of two individuals *i* and *j* originates from the polygenic random effects *g*_*i*_ and *g*_*j*_. The random effects depend on the relatedness of both individuals which can be expressed in terms of *G*_*ij*_ varying between zero and one. This implies that the polygenic contribution to the phenotype ranges from “independent” to “identical” for a pair of individuals. We assume that residuals are uncorrelated between observations and distributed as multivariate normal $\mathbf {e} \sim \mathrm {N}_{n}(0,\sigma ^{2}_{\mathrm {e}}\mathbf {I})$ with certain variance $\sigma ^{2}_{\mathrm {e}}$ and identity matrix **I**. The heritability of **y**=(*y*_1_,*y*_2_,…,*y*_*n*_) can be expressed through $R^{2}_{\mathrm {h}}=\sigma ^{2}_{\mathrm {g}}/\left (\sigma ^{2}_{\mathrm {g}}+\sigma ^{2}_{\mathrm {e}}\right)$.

Ignoring relatedness results in the following simpler model to be fitted to the data:

2 $$ y_{i}=\beta_{1}+\beta_{2} s_{i}+\epsilon_{i}  $$

assuming uncorrelated residuals ***ε***=(*ε*_1_,*ε*_2_,…,*ε*_*n*_) only. We aim at deriving analytical formulae for the expectation and variance of $\hat \beta _{2}$ given the true model, i.e. we analyse the impact of relatedness on the estimates obtained with Eq. (2).

After some calculations (Additional file 2: Section 2.2), it follows that the expected value $\mathrm {E}(\hat \beta _{2})=b_{2}$ is not biased by relatedness irrespective of its structure. However, the variance of $\hat \beta _{2}$ is affected:

3 $$\begin{array}{*{20}l} \mathrm{V}(\hat\beta_{2})= & \frac{\sigma^{2}_{\mathrm{e}}}{\sum^{n}_{i=1}{(s_{i}-\bar{s})^{2}}}\frac{1}{1-R^{2}_{\mathrm{h}}}\\ & \left(1+R^{2}_{\mathrm{h}}\frac{\sum^{n}_{i=1}\sum^{n}_{j \neq i=1}{G_{ij}(\bar{s}^{2}-2\bar{s}s_{i}+s_{i} s_{j})}}{\sum^{n}_{i=1}{(s_{i}-\bar{s})^{2}}}\right). \end{array} $$

Without heritability, i.e. $R^{2}_{\mathrm {h}}=0$, the phenotypes for all pairs of individuals are uncorrelated and the last two terms of Eq. (3) simplify to 1. In this case, we obtain

$$ V_{\upbeta}=\frac{\sigma^{2}_{\mathrm{e}}}{\sum^{n}_{i=1}{(s_{i}-\bar{s})^{2}}}. $$

This variance is equivalent to the variance of the standard linear model as shown in [16]. For the last term of $\mathrm {V}(\hat \beta _{2})$ in Eq. (3), we define the inflation factor

4 $$ \lambda=1+R^{2}_{\mathrm{h}}\frac{\sum^{n}_{i=1}\sum^{n}_{j \neq i=1}{G_{ij}(\bar{s}^{2}-2\bar{s}s_{i}+s_{i}s_{j})}}{\sum^{n}_{i=1}{(s_{i}-\bar{s})^{2}}}  $$

which depends on the heritability $R^{2}_{\mathrm {h}}$ and the pairwise relatedness matrix **G**. Using *λ*, $\mathrm {V}(\hat \beta _{2})$ can be rewritten as

5 $$ \mathrm{V}(\hat\beta_{2})=\frac{\lambda}{1-R^{2}_{\mathrm{h}}}V_{\upbeta}.  $$

As we will see in the “Hypothesis testing” section, the empirical variance of the effect estimate is also inflated by factor $1/\left (1-R^{2}_{\mathrm {h}}\right)$. Hence, this factor is cancelled out when estimating the corresponding *T* statistic.

### Expected variance inflation

An approximation formula for *λ* can be obtained by separately deriving the expectations of the numerator and denominator of Eq. (4) as shown in Additional file 2: Section 3.2:

6 $$ \lambda'=1+R^{2}_{\mathrm{h}}\frac{G_{2}-\frac{2}{n}G_{\mathrm{r}}}{n-1}  $$

where

$$\begin{array}{@{}rcl@{}} G_{2} & = & \sum\limits^{n}_{i=1}\sum\limits^{n}_{j \neq i=1}{G^{2}_{ij}},\\ G_{\mathrm{r}} & = & \sum\limits^{n}_{i=1}\left(\sum\limits^{n}_{j \neq i=1}{G_{ij}}\right)^{2} \end{array} $$

correspond to the sum of squared elements and the sum of the squared row sums of **G**, respectively. Approximating E(*λ*) by *λ* ^′^ is valid if the number *n* of observations is large and the mean relatedness

$$ \bar{G}=\frac{1}{n(n-1)}\sum\limits^{n}_{i=1}\sum\limits^{n}_{j \neq i=1}{G_{ij}} $$

is small. Interestingly, Eq. (6) is independent of the allele frequency explaining the empirical observations of [1, 14]. For details, see Additional file 2: Section 3.2.

### Relationship between heritability and inflation

There are some useful transformations of Eq. (6): If *λ* ^′^ is available for a specific heritability $R^{2}_{\mathrm {h}}$, it is easy to derive the inflation *λ*t′ for an alternative heritability $R^{2}_{\mathrm {t}}$ given the same relatedness structure. As can be seen from Eq. (6), it holds that

7 $$\begin{array}{*{20}l}  R^{2}_{\mathrm{t}} &=& \frac{\lambda'_{\mathrm{t}}-1}{\lambda'-1}R^{2}_{\mathrm{h}}, \end{array} $$

8 $$\begin{array}{*{20}l}  \lambda'_{\mathrm{t}} &=& 1+(\lambda'-1)\frac{R^{2}_{\mathrm{t}}}{R^{2}_{\mathrm{h}}}. \end{array} $$

See also Additional file 2: Section 3.3.

### Example family structures

Using Eq. (6), inflation *λ* ^′^ can be estimated for arbitrary family structures. As an example, assume a family study with *f* families with one father per family. Each father is mated with *m* mothers and each mother has *c* children. Then, the number of samples is *n*=(*c* *m*+*m*+1)*f*. Given these relationships as relatedness matrix **G**, inflation *λ* ^′^ can be explicitly calculated by

9 $$\begin{array}{*{20}l} \lambda'_{f;m;c}= & 1+R^{2}_{\mathrm{h}} \left[ \left(\left(c^{3}+c^{2}\right)f-2c^{3}\right)m^{3}\right.\\ &+\left(\left(3c^{3}+16c^{2}+12c\right)f-4c^{3}-16c^{2}\right)m^{2}+\\ & \left.\left(\left(3c^{2}+12c\right)f-2c^{3}-16c^{2}-8c\right)m\right]/\\ &\left[ (16c^{2}+32c+16)fm^{2}+ \right.\\ & \left.\left(\left(32c+32\right)f-16c-16\right)m+16f-16\right]. \end{array} $$

The formula is implemented in an R script (see Additional file 1). The special case of *m*=1, *c*=1 corresponds to trios in which Eq. (9) simplifies to

10 $$ \lambda'_{f;1;1}=1+R^{2}_{\mathrm{h}} \frac{f-1}{3f-1}.  $$

Another example is a study with an increased number of pairwise relationships (*m*=2, *c*=3) where Eq. (9) simplifies to

11 $$ \lambda'_{f;2;3}=1+R^{2}_{\mathrm{h}} \frac{243f-314}{216f-24}.  $$

Details of these formulae are provided in Additional file 2: Section 3.4 and Additional file 3.

### Hypothesis testing

Assume we observe phenotypes **y** and SNP genotypes **s** obeying Eq. (1). We are interested whether the phenotype is associated with the SNP. For the simplified regression model in Eq. (2), this corresponds to testing the null hypothesis of *β*_2_=0. Thus, the test statistic $T=\hat \beta _{2}/S_{\upbeta }$ as presented in [17] is evaluated. $S^{2}_{\upbeta }$ denotes the empirical variance estimate of $\hat \beta _{2}$. Evaluating the distribution of the test statistic under the null hypothesis is required for assessing the type I error. The distribution of the test statistic under the alternative hypothesis is required for calculating the power of the test. In reference to Additional file 2: Section 5.1, the effect estimate $\hat \beta _{2}$ is normally distributed, and, if the variance of $S^{2}_{\upbeta }$ is small, one can replace $S^{2}_{\upbeta }$ by its expected value $\mathrm {E}\left (S^{2}_{\upbeta }\right)$. This implies that *T* is approximately normally distributed with expectation and variance as follows

$$\begin{array}{@{}rcl@{}} T \sim \mathrm{N}\left(\frac{\mathrm{E}(\hat\beta_{2})}{\sqrt{\mathrm{E}\left(S^{2}_{\upbeta}\right)}},\frac{\mathrm{V}(\hat\beta_{2})}{\mathrm{E}\left(S^{2}_{\upbeta}\right)}\right). \end{array} $$

Assuming the null hypothesis, one obtains $\mathrm {E}(\hat \beta _{2})=0$. Further, using $\mathrm {V}(\hat \beta _{2})=\lambda V_{\upbeta }/\left (1-R^{2}_{\mathrm {h}}\right)$ as shown in Eq. (5) and $\mathrm {E}\left (S^{2}_{\upbeta }\right) \approx V_{\upbeta }/\left (1-R^{2}_{\mathrm {h}}\right)$ as given in Additional file 2: Section 4.2, the distribution of *T* can be calculated:

12 $$ T \sim \mathrm{N}(0,\lambda).  $$

See also Additional file 2: Section 5.2.

Considering the alternative hypothesis, it holds that $\mathrm {E}(\hat \beta _{2})=b_{2}$ and $\mathrm {E}\left (S^{2}_{\upbeta }\right) \approx V_{\upbeta }/\left (1-R^{2}_{\mathrm {h}}\right)$ in analogy to the null hypothesis. In the following, we assume a fixed explained variance of the SNP $R^{2}_{\mathrm {s}}$. Thus, the SNP effect is described by only one parameter. Alternatively, if specifying a fixed SNP effect *b*_2_, test statistics would also depend on the allele frequency, i.e. two parameters would be required. For a given $R^{2}_{\mathrm {s}}$, it holds that

13 $$ \mathrm{E}(T) \approx \sqrt{(n-1)R^{2}_{\mathrm{s}}}=\mu  $$

as shown in Additional file 2: Section 5.3. Finally, an approximation of the distribution of *T* under the alternative hypothesis can be derived:

14 $$ T \sim \mathrm{N}(\mu,\lambda).  $$

Here, caused by relatedness, the empirical variance of the effect estimate $\mathrm {E}\left (S^{2}_{\upbeta }\right)$ is deflated compared to $V_{\upbeta }/\left (1-R^{2}_{\mathrm {h}}\right)$ by a certain factor *ν* as shown in Additional file 2: Section 4.2.

Further, assume F_N_(*x*|*μ*,*σ* ^2^) is the cumulative distribution function of the normal distribution with expectation *μ* and variance *σ* ^2^. Given the quantile *z*_*α*/2_ of the standard normal distribution corresponding to a two-sided test with significance level *α*, the type I error of the test applying Eq. (12) can be derived

15 $$ \text{err} = 2\mathrm{F}_{\mathrm{N}}(z_{\alpha/2}|0,\lambda).  $$

Similarly, the power of the test applying Eq. (14) is

16 $$ \text{pwr} = \mathrm{F}_{\mathrm{N}}\left(z_{\alpha/2}|\mu,\lambda\right)+1- \mathrm{F}_{\mathrm{N}}\left(-z_{\alpha/2}|\mu,\lambda\right).  $$

### Genomic control

Genomic control [12] is a simple and often used method to correct for variance inflation. Given a sample of *n* realisations $\hat {T}_{1},\hat {T}_{2},\dots,\hat {T}_{n}$ of *T* under the null hypothesis, an estimate of *λ* according to Additional file 2: Section 6 is

$$ \hat\lambda=\frac{\text{median}\left(\hat{T}^{2}_{1},\hat{T}^{2}_{2}\dots,\hat{T}^{2}_{n}\right)}{0.456}. $$

Genomic control correction is performed by calculating $T_{\text {gc}}=T/\sqrt {\hat \lambda }$ and using *T*_gc_ as new test statistic. Correcting the variance inflation of *T* under the null hypothesis (see Eq. (12)), the test statistic *T*_gc_ is approximately standard normally distributed:

17 $$ T_{\text{gc}} \sim \mathrm{N}(0,1).  $$

Since

18 $$ \text{err}_{\text{gc}}=2\mathrm{F}_{\mathrm{N}}(z_{\alpha/2}|0,1)=\alpha,  $$

the type I error of the test is preserved.

In contrast, correction of the alternative statistic *T* distributed as shown in Eq. (14) yields

19 $$ T_{\text{gc}} \sim \mathrm{N}\left(\frac{\mu}{\sqrt{\hat\lambda}},1\right).  $$

Thus, genomic control correction reduces the expectation of the test statistic, and with it, the power of the test in comparison to Eq. (16) unless *λ* is close to 1:

20 $$ \text{pwr}_{\text{gc}} = \mathrm{F}_{\mathrm{N}}\left(z_{\alpha/2}\Big|\frac{\mu}{\sqrt{\hat\lambda}},1\right)+1- \mathrm{F}_{\mathrm{N}}\left(-z_{\alpha/2}\Big|\frac{\mu}{\sqrt{\hat\lambda}},1\right).  $$

### Samples

To apply our equations to real data, we consider HapMap CEU (CEPH (Centre d’Etude du Polymorphisme Humain) from Utah) trio data for two reasons. First, these genotype data is freely accessible and well understood so that our results can easily be reproduced. Secondly, the relatedness structure is simple in order to promote understanding of our equations. A simple relatedness structure also supports simulation of genotype data to obtain results under different settings, e.g. increased sample size. Filtering of HapMap SNPs and samples prior to analysis is described in Additional file 4. A matrix of pairwise relatedness estimates for all HapMap CEU samples is provided as Additional file 5. In summary, 1,020,215 SNPs measured in 129 HapMap samples belonging to 43 trios were available for analysis. Additional file 6 contains a detailed list of samples and the reason for exclusion where applicable, whereas Additional file 7 provides the list of SNP identifiers used for analysis. The Perl script provided as Additional file 8 together with the sample list in Additional file 6 and the SNP list in Additional file 7 can be used for converting the HapMap CEU data [18] to a CSV (comma separated values) file which is further analysed.

Furthermore, we analysed a sample of the Sorbs who are an ethnic minority in Germany with putative genetic isolation [13, 19]. The Sorbs sample is characterised by a complex relatedness structure and therefore suitable for analysis of variance inflation. As done in [13], 471,012 autosomal SNPs were filtered for call rate < 95*%*, deviation from Hardy-Weinberg equilibrium with *p*<10^−6^ and platform association with *p*<10^−7^. After filtering, 424,476 SNPs measured in 977 samples were available for analysis.

Finally, synthetic genotypes were simulated for three studies each consisting of *f* families with one father per family, *m* mothers per father and *c* children per mother as described in Additional file 2: Section 3.4. In order to evaluate the results obtained for the HapMap data, a study (SFS1, synthetic family study 1) was simulated for *n*=129 samples with parameter set *f*=43, *m*=1, *c*=1. For the second study (SFS2), the relatedness structure was kept similar but the sample size was increased to *n*=999, i.e. the parameter set was *f*=333, *m*=1, *c*=1. For stronger relationships but the same *n*=999 samples, we simulated a third study (SFS3) with parameter set *f*=111, *m*=2, *c*=3. For all synthetic studies, we sampled 110,000 SNPs where the reference allele of each SNP was drawn from a beta distribution (shape *a*=0.5, shape *b*=0.5).

### Simulation

For simulation and analysis of the results, we used the statistical software package R [20]. The script is provided as Additional file 1. Instead of sampling SNPs for a synthetic family study, genotypes provided as CSV file can also be loaded and analysed utilising this R script. The HapMap and Sorbs genotype data were analysed in this way. In any case, a random subset of 100,000 non-monomorphic SNPs was selected for all studies. The R script was also used to estimate pairwise relatedness according to Wang [15], to calculate the variance inflation *λ* given the SNP genotypes as presented in Eq. (4) averaged over all SNPs and to calculate the expected inflation *λ* ^′^ based on estimated relationships as shown in Eq. (6). Further, the R script supports simulation of phenotypes under the null and alternative hypothesis assuming Eq. (1) for empirical verification of the test statistics as presented in Eqs. (12) and (14), respectively. Empirical values of the statistics were derived by simulations as follows: For each SNP, phenotypes are drawn repeatedly from a multivariate normal distribution where the expectation depends on the SNP if simulating alternative hypotheses or is independent of it for simulating null hypotheses. These simulated test statistics were averaged over phenotype realisations and the empirical variance was estimated to assess inflation due to relatedness. The resulting mean test statistics and their empirical variances were averaged over SNPs and a standard deviation was calculated to control sampling errors. Due to the computational burden, simulations were restricted to 1000 phenotype realisations per SNP and a random subset of 1000 SNPs.

## Results

### Variance inflation for examples of relatedness

We apply the formulae derived in the “Methods” section to assess and compare variance inflation between different scenarios of relatedness structure and heritability. Given the genotypes of a SNP **s**, the estimated relatedness matrix **G** and the heritability $R^{2}_{\mathrm {h}}$ one can calculate the variance inflation based on Eq. (4).

**Fig. 1:**
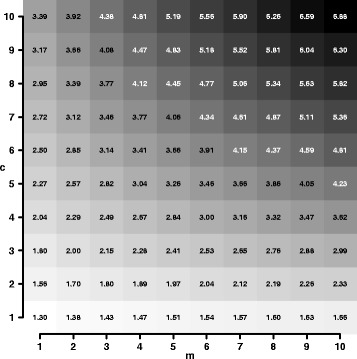
Expected variance inflation for synthetic family studies. The figure presents the expected variance inflation *λ* *f*;*m*;*c*′ for heritability $R^{2}_{\mathrm {h}}=0.9$ and family studies with varying numbers of mothers *m* and children *c*, each between 1 and 10, and with a total of about *n* =1000 individuals. The background colour corresponds to the values presented and ranges from white for the minimum to black for the maximum inflation

Different relatedness structures result in different degrees of variance inflation. We demonstrate this on an example of a synthetic family study consisting of *f* families with one father per family, *m* mothers and *c* children. Further, assume that each study comprises the same number *n* of individuals but differs in *c* and *m*. Therefore, we set *f*=floor(*n*/(*c* *m*+*m*+1)) (“floor” returns the largest integer not greater as the argument) and estimate the expected variance inflation of the effect estimate by evaluating Eq. (9). Figure 1 shows the expected inflation *λ* *f*;*m*;*c*′ for heritability $R^{2}_{\mathrm {h}}=0.9$ and different settings of *m* and *c* resulting in the same sample size *n*=1000. For example, a trio study with *f*=333, *m*=1 and *c*=1 (*n*=999) results in *λ*333;1;1′=1.3. This value can also be obtained via Eq. (10). A more extreme example is a family study with *f*=111, *m*=2 and *c*=3 (*n*=999) which results in *λ*111;2;3′=2 (see also Eq. (11)). Inflation *λ* ^′^ also depends on sample size, but notable differences can only be observed for small sample sizes (i.e. *n*<100).

For a random subset of 100,000 non-monomorphic SNPs, we estimated the variance inflation for the real HapMap trio data, the Sorbs data and the above mentioned synthetic family studies SFS1 (corresponding to HapMap study), SFS2 (corresponding to trios with a larger sample size of *n*=999) and SFS3 (corresponding to the same sample size as SFS2 but a higher average relatedness). Results for $R^{2}_{\mathrm {h}}=0.9$ are presented in Table 1. The empirical variance inflation *λ* is smallest for HapMap and SFS1, the latter two are in well agreement as expected. The higher sample size for SFS2 results in slightly higher inflation. The Sorbs inflation is even higher than for SFS2. As expected, SFS3 shows the strongest inflation. Using *λ* ^′^ instead of *λ* results in slightly higher values due to the Taylor expansion used to derive Eq. (6) (see Additional file 2: Section 3.2). But the difference is without practical relevance. Restricting to minor allele frequencies > 10*%* improves the agreement (see Table 1 column $\bar \lambda _{10\%}$). The expected variance inflation *λ* ^′^ calculated from the estimated relatedness matrix agrees well with *λ* *f*;*m*;*c*′ calculated from true relationships. Of note, if heritability $R^{2}_{\mathrm {t}}$ drops below 10% for HapMap, Sorbs, SFS1 and SFS2 according to Eq. (7), inflation becomes irrelevant (*λ*t′<1.05, see Table 1 for details). However, inflation for the extreme situation of study population SFS3 is still *λ*t′=1.11 as calculated with Eq. (8).

### Numerical validation of test statistics

The distributions of the test statistic *T* in Eqs. (12) and (14) are approximations due to the approximation of the variance estimate. To empirically verify these approximations, we simulated multivariate normally distributed phenotypes and fitted a linear model afterwards. We analysed the same five study populations as in the previous section and again assumed $R^{2}_{\mathrm {h}}=0.9$. Results are presented in Table 2 for the null hypothesis and Table 3 for the alternative hypothesis. The expectation and empirical variance of *T* was averaged over SNPs. As expected, the expectation of *T* under the null hypothesis is close to zero for all studies (Table 2). The expectation under the alternative is close to its theoretical value *μ* calculated via Eq. (13) (Table 3), i.e. no relevant biases were observed for *T* under both hypotheses. However, the variance of *T* is slightly overestimated in comparison to the derived *λ* values presented in Table 1 (compare $\bar {S}^{2}$ of Tables 2 and 3 with $\bar \lambda $ of Table 1). The difference is more pronounced for the studies with small samples sizes, i.e. HapMap and SFS1. For larger studies, the difference is without practical importance. Although the empirical variance of the effect estimate is deflated by factor *ν* (see Additional file 2: Section 4.2 and Table 2), this deflation is close to 1 in our data, and again, is without practical relevance.

**Table 2:** Simulation results for the test statistic *T* under the null hypothesis

Study	$\bar {T}$	$\bar {S}^{2}$	*ν*
HapMap	0.002 (0.037)	1.330 (0.096)	0.992
SFS1	-0.000 (0.037)	1.321 (0.107)	0.992
SFS2	-0.001 (0.037)	1.309 (0.076)	0.999
Sorbs	-0.001 (0.037)	1.412 (0.144)	0.999
SFS3	0.001 (0.043)	2.015 (0.166)	0.997

The test statistics $\bar {T}$ averaged over replicates and SNPs and the average of the empirical variances $\bar {S}^{2}$ are compared between HapMap, SFS1 (synthetic family study 1), SFS2, Sorbs and SFS3 assuming the null hypothesis and $R^{2}_{\mathrm {h}}=0.9$. Standard deviations are presented in parentheses. We further provide an estimate of the deflation factor *ν* for the empirical variance of the effect estimate

**Table 3:** Simulation results for the test statistic *T* under the alternative hypothesis

Study	$\bar {T}$	$\bar {S}^{2}$	*μ*
HapMap	1.619 (0.037)	1.343 (0.095)	1.600
SFS1	1.619 (0.036)	1.336 (0.112)	1.600
SFS2	4.472 (0.036)	1.330 (0.076)	4.468
Sorbs	4.420 (0.039)	1.432 (0.148)	4.418
SFS3	4.479 (0.046)	2.030 (0.162)	4.468

The test statistics $\bar {T}$ averaged over replicates and SNPs and the average of the empirical variances $\bar {S}^{2}$ are compared between HapMap, SFS1 (synthetic family study 1), SFS2, Sorbs and SFS3 assuming the alternative hypothesis with $R^{2}_{\mathrm {s}}=0.02$ and heritability $R^{2}_{\mathrm {h}}=0.9$. Standard deviations are presented in parentheses. We further provide the expected value *μ* of the test statistic *T*

### Examples of inflation factors

Since heritability and relatedness structure directly translate into inflation factors, we study the latter in the following in more detail. To study type I error and power of the tests, we consider four different inflation scenarios *λ*=1, i.e. no inflation, and *λ*=1.05,1.3 and 2. For example, any study comprising unrelated individuals results in about *λ*=1, whereas our study populations SFS1 with $R^{2}_{\mathrm {h}}=0.15$, SFS2 and SFS3 with $R^{2}_{\mathrm {h}}=0.9$ result in about *λ*=1.05, 1.3 and 2, respectively. See also Table 1 for the latter three scenarios.

### Impact of inflation on type I error

**Fig. 2:**
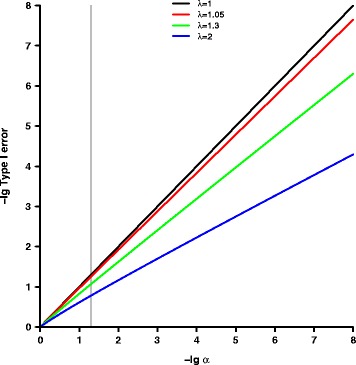
Comparison of type I errors with respect to different degrees of variance inflation. The figure provides a comparison of type I errors dependent on the significance level *α* without variance inflation *λ*=1 and variance inflation with *λ*=1.05, 1.3 and 2. The negative common logarithm is presented for *α* as well as the type I error. The grey vertical line corresponds to a significance level of *α*=0.05

In the situation of statistical testing, the variance of *T* under the null hypothesis is relevant for the type I error. Its inflation originates from heritability $R^{2}_{\mathrm {h}}$ and the family structure as shown in Eq. (4). Variance inflation *λ* impacts the distribution of the test statistic under the null hypothesis as shown in Eq. (12) and affects the type I error of the test as depicted in Eq. (15). In Fig. 2, we present the type I error dependent on the significance level without inflation *λ*=1 and inflation with *λ*=1.05,1.3 and 2 as in the above mentioned scenarios. Type I error for *λ*=1.05 is similar to *λ*=1 justifying the 1.05 threshold typically applied to ignore inflation. However, the type I error increases rapidly with increasing inflation.

### Impact of inflation on power

**Fig. 3:**
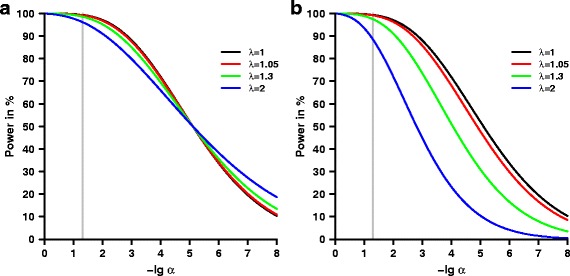
Comparison of power with respect to different degrees of variance inflation. Both figures provide a comparison of power in percent dependent on the significance level *α* without variance inflation *λ*=1 and variance inflation with *λ*=1.05, 1.3 and 2. Figure **a** corresponds to the uncorrected test statistic, whereas Figure **b** refers to the test statistic after genomic control. The negative common logarithm is presented for *α*. The grey vertical line corresponds to a significance level of *α*=0.05. An explained variance of $R^{2}_{\mathrm {s}}=0.02$ was assumed. Sample size was set to *n*=1000

For calculating the power, expectation and variance of *T* under the alternative is required. As shown in Eq. (4), variance inflation depends on heritability $R^{2}_{\mathrm {h}}$ and the family structure. Similar to the null hypothesis, variance inflation *λ* impacts the distribution of the test statistic under the alternative as shown in Eq. (14) and affects the power of the test (Eq. (16)). The expectation of *T*, see Eq. (13), depends on the sample size *n* and the explained variance by the SNP $R^{2}_{\mathrm {s}}$. We assume *n*=1000 and $R^{2}_{\mathrm {s}}=0.02$ resulting in an expectation of the test statistic of $\mu =\sqrt {(n-1)R^{2}_{\mathrm {s}}} \approx 4.47$. For this expectation, we present Fig. 3 a showing the dependence of power, see Eq. (16), on the significance level for *λ*=1 (no inflation) and *λ*=1.05,1.3 and 2. The power for *λ*=1.05 is similar to *λ*=1, indicating again that this inflation is negligible for practical purposes. The difference is more pronounced for the other power curves with *λ*>1.05. Irrespective of the variance of the test statistic, the power curves are intersecting at 50%. For the selected expectation, this corresponds to −lg(*α*)≈5.11 (“ lg” refers to the common logarithm with base 10). Thus, for smaller significance levels, the power increases with increasing inflation while the opposite occurs for larger significance levels.

### Correction with genomic control

In case of inflation, an often applied method of correction is genomic control. If this correction is applied in the situation of relatedness, the distribution of the test statistic (Eq. (17)) under the null hypothesis is approximately standard normal. This implies that the type I error *α* (Eq. (18)) is preserved. In contrast, correcting the test statistic by the inflation factor reduces the expectation (Eq. (19)) under the alternative hypothesis which in turn reduces the power (Eq. (20)) of the test. In Fig. 3 b, we provide the power dependent on the significance level after genomic control without inflation *λ*=1 and with inflation *λ*=1.05, 1.3 and 2. Comparing Fig. 3 a and b, power loss of genomic control increases rapidly with increasing *λ*. Thus, genomic control cannot be recommended for inflations *λ*>1.05 induced by relatedness.

## Discussion

Relatedness induces a dependency structure to phenotypic data, and therefore, needs to be addressed appropriately in genetic association studies. However, the impact of relatedness on key statistical properties is insufficiently studied and major insights rely on simulation studies only. Here, we provide a full theory of the impact of relatedness on linear regression analysis of a quantitative phenotype. We derive analytical formulae of test statistics and provide a simple approximate formula of the dependence of variance inflation on the relatedness structure. We studied the impact of relatedness on type I error and power and confirmed a number of phenomena observed in simulation studies. Moreover, we showed that genomic control cannot be recommended to deal with relatedness-induced inflation. All formulae were implemented in an R script provided as supplement (Additional file 1).

First, we derived formulae of the impact of relatedness on effect estimates and variances of a linear regression model. We proved that the expectation is unbiased in agreement with [1, 11] who observed this fact on the basis of simulation studies. We derived an approximation formula of the variance inflation given the relatedness and the heritability of the phenotype. We also proved that the standard error of the effect estimate is underestimated if applying the standard linear model. This is reflected by the deflation factor *ν* derived in Additional file 2: Section 4.2. Again, this issue was observed by [1] on the basis of a simulation study.

We estimated this variance inflation for “real” genotype data obtained from HapMap trios and the Sorbs and for synthetic genotypes of three different family studies of varying degree of relatedness. For a heritability of 90%, we showed that there is a relevant inflation for all of these studies. In contrast, if heritability drops below 10%, the inflation is only relevant in the extreme situation of study population SFS3. See also Additional file 9 for additional results of scenarios with varying degree of heritability.

The polygenic effect was modelled via a multivariate normal distribution with the relatedness matrix as covariance matrix. Alternatively, the polygenic effect could be modelled by single markers as proposed by Zhang et al. [3]. Results are similar even for small numbers of SNPs contributing to the polygenic effect (see Additional file 10).

For analysis, we utilised relatedness estimates obtained from genomic data rather than estimates obtained from pedigree data. First, correct pedigree data are difficult to assess especially for non-family studies or studies with cryptic relatedness as observed in isolated populations, e.g. the Sorbs [13]. Second, [5, 14] argued that estimates from marker data reflect true genetic relationships better then estimates from even a correct pedigree. In contrast to [5] who applied kinship estimates as presented in [21], we estimated pairwise relatedness with the method proposed by Wang [15]. The latter has several advantages as correction for allele frequency estimates. Otherwise, relatedness estimates could be biased [15, 21], see also Fig. 1 in Additional file 11. However, in our hands using the kinship matrix [5, 21] or the IBS(identical by state)-based matrix [4, 22] as alternative estimators, this has little impact on the inflation results (see Additional file 11). Further, the method in [15] results in a diagonal of the estimated relatedness matrix identical to 1 which is required for our derivations in Additional file 2: Section 2.2.

In general, inflation depends on the allele frequency of a SNP. However, considering our approximation formula Eq. (6), this dependency can be neglected if the sample size is sufficiently large and the average relatedness is small. This explains corresponding empirical observations of [1, 14].

As different combinations of relatedness structure and heritability yield the same variance inflation, we further focused on different degrees of variance inflation to study type I error and power. For this purpose, we derived an analytical approximation of the test statistic given the variance inflation. The approximation was successfully verified in a simulation study.

We showed analytically that the type I error increases with inflation. With our formula, we could confirm the empirical observation of [1, 11] that type I error of the test increases with higher heritability and stronger relationships. Similarly, [9] observed an inflated type I error when the family structure is ignored.

A major result of our study is that the power increases with increasing inflation if the significance level is small while the opposite occurs for larger significance levels. We already observed this phenomenon in a previously published simulation study [13]. This explains a number of contrary empirical observations presented in the literature, e.g. [1, 9] noted that the power of the test is reduced when ignoring the family structure. However, [11] observed similar power irrespective whether accounting for the family structure or not. By our formula, we could show that the power could be either increased or decreased under inflation in dependence on the underlying significance threshold.

Our formulae can also be applied to compare the impact of family structures between studies. Power and type I error were analysed previously in [1, 5] for a nuclear pedigree (NP) of 1011 individuals belonging to 337 sib trios. Applying our formulae (Additional file 3), this family structure results in an inflation factor of 1.45 for $R^{2}_{\mathrm {h}}=0.9$. Interestingly, the same value was observed for the Sorbs sample.

Since genomic control is an often applied method to correct for inflated test statistics, we studied its results in the situation of relatedness-induced inflation. We could show that genomic control maintains the correct type I error which is in line with [5, 12]. However, we also showed that genomic control seriously impairs power. This was acknowledged by [12] for increased inflation and by [5] for higher heritability and stronger relationships. According to our results, genomic control cannot be recommended to deal with inflation due to relatedness. One has to remark that genomic control was originally developed to correct for population stratification [23, 24]. In contrast to other studies [12, 14, 21], we did not consider additional population structure here. Results for selected settings of heritability and explained variance of the SNP are presented in the paper. More scenarios can be easily analysed using our R script provided as Additional file 1.

The properties of various correction methods as well as simple linear regression are compared in [10]. Here, we investigated the linear model in detail, provided an easy to apply approximation formula of the impact of relatedness on variance inflation and identified scenarios where simple linear regression analysis is still valid. We agree with Aulchenko [14] that a variance inflation below 1.05 is negligible regarding power and type I error. If variance inflation is larger, we advice to apply methods which explicitly account for relatedness, e.g. by mixed model analysis [1, 5, 9, 25–27]. Nonetheless, these models need to be carefully applied due to several pitfalls [28]. For a summary of correction methods and software tools, see also [29].

## Conclusions

We developed approximation formulae to study the impact of relatedness on type I error and power. We could prove a number of empirical observations made in simulation studies. Stronger relatedness as well as higher heritability result in increased variances of the effect estimates of simple linear regression analyses. As a consequence, type I error rates are generally inflated. The behaviour of power is more complicate since relatedness could either increase or reduce it in dependence on the effect size of a SNP, the heritability of the phenotype and the significance threshold. Genomic control cannot be recommended to deal with relatedness-induced inflation. Variance inflation below 1.05 can be safely ignored, i.e. simple linear regression analysis is still appropriate in this case.

## Additional files

Additional file 1 R script for simulation. This R script supports simulation of synthetic genotypes for a family study. Instead of genotype simulation, genotypes can also be loaded from a CSV file. Allele frequencies are calculated, monomorphic SNPs are filtered and pairwise relatedness is estimated. Given SNP genotypes and a value for the heritability, variance inflation *λ*is calculated. Additionally, the expected *λ* ^′^ is estimated. Finally, the script simulates phenotypes under the null and alternative hypothesis and provides results regarding the *T* statistic. The R library “mvtnorm” is required for sampling multivariate normally distributed phenotypes. Parameters can be modified to simulate different scenarios. However, the number of samples, the number of SNPs and the number of phenotype realisations per SNP should be limited to reduce the computational burden. For example, running the script on an Intel Xeon X5560 CPU (2.80 GHz) for synthetic family study 3 (SFS3) with parameter set *f*=111, *m*=2, *c*=3 (*n*=999), 100000 SNPs, 1000 phenotype realisations per SNP and 1000 SNPs required 8.3 GB RAM and took < 1 min for genotype sampling, 8 min for estimation of pairwise relatedness, 21 min for *λ* estimation and about 2.5 h for each of the phenotype simulations under the null and alternative hypothesis, respectively. (R 6 kb)

Additional file 2 Theoretical background. This file provides the theoretical background and derivations of equations presented in the manuscript. (PDF 231 kb)

Additional file 3 Maxima script for deriving expected variance inflation. This script can be used with Maxima [30] for deriving formulae for the expected variance inflation *λ* *f*;*m*;*c*′ for synthetic family studies. (WXM 1 kb)

Additional file 4 Preparation of HapMap data. This document provides details regarding the filtering of samples and SNPs of the HapMap data. (PDF 97 kb)

Additional file 5 Pairwise relatedness estimates of HapMap samples. This file contains a matrix of pairwise relatedness estimates resulting from the preliminary analysis of 174 HapMap CEU samples. Sample identifiers for the pair of individuals under consideration are given in the first row and in the first column, respectively. A value of -1 occurs if pairwise relatedness could not be estimated because of disjoint SNP sets. (CSV 571 kb)

Additional file 6 Sample selection of HapMap genotype data. This file provides annotations for 174 HapMap CEU samples. The columns FID (family identifier), IID (individual identifier), dad, mom, sex (1=male, 2=female), pheno (always 0), population (always CEU) correspond to the columns of relationships_w_pops_121708.txt filtered for CEU samples as provided by HapMap. The column ctr contains a unique trio identifier and equals NA when the sample does not belong to a complete trio family. The reason for exclusion is provided where applicable, otherwise NA is stated and the sample is included in our study. (CSV 8 kb)

Additional file 7 SNP selection of HapMap genotype data. This file contains a list of HapMap SNP identifiers used for our analyses. rsid (reference SNP identifier) refers to the first column of the genotype data files as provided by HapMap. (CSV 10000 kb)

Additional file 8 Perl script for converting HapMap genotype data. This Perl script requires the sample list of Additional file 6, the SNP list of Additional file 7 and HapMap raw data. The HapMap project website is not available anymore, however, genotype data can still be retrieved from ftp://ftp.ncbi.nlm.nih.gov/hapmap/genotypes/2010-08_phaseII+III/. The converted genotypes are saved in a CSV file. Folder and file locations must be adapted before running the script. Running the script on an Intel Xeon X5560 CPU (2.80 GHz) required 800 MB RAM and took about 5 minutes. (PL 2 kb)

Additional file 9 Comparison of different degrees of heritability. This file contains additional tables with inflation results for different degrees of heritability. (PDF 75 kb)

Additional file 10 Comparison of methods for modelling the polygenic effect. This file provides additional tables with inflation results for different polygenic models. (PDF 67 kb)

Additional file 11 Comparison of different relatedness estimators. This document summarizes different methods for estimating relatedness, presents corresponding inflation results and shows the impact of small allele frequencies on relatedness estimates. (PDF 140 kb)

## Abbreviations

CEU: CEPH (Centre d’Etude du Polymorphisme Humain) from Utah
CSV: Comma separated values
IBD: Identical by descent
IBS: Identical by state
SFS: Synthetic family study
SNP: Single nucleotide polymorphism

## References

[1] Aulchenko YS, de Koning DJ, Haley C (2007). Genomewide rapid association using mixed model and regression: a fast and simple method for genomewide pedigree-based quantitative trait loci association analysis. Genetics.

[2] Boerwinkle E, Chakraborty R, Sing CF (1986). The use of measured genotype information in the analysis of quantitative phenotypes in man. I, Models and analytical methods. Ann Hum Genet.

[3] Zhang YM, Mao Y, Xie C, Smith H, Luo L, Xu S (2005). Mapping quantitative trait loci using naturally occurring genetic variance among commercial inbred lines of maize (Zea mays L.). Genetics.

[4] Yu J, Pressoir G, Briggs WH, Vroh Bi I, Yamasaki M, Doebley JF, McMullen MD, Gaut BS, Nielsen DM, Holland JB, Kresovich S, Buckler ES (2006). A unified mixed-model method for association mapping that accounts for multiple levels of relatedness. Nat Genet.

[5] Amin N, van Duijn CM, Aulchenko YS (2007). A genomic background based method for association analysis in related individuals. PLoS ONE.

[6] Wang SB, Feng JY, Ren WL, Huang B, Zhou L, Wen YJ, Zhang J, Dunwell JM, Xu S, Zhang YM (2016). Improving power and accuracy of genome-wide association studies via a multi-locus mixed linear model methodology. Sci Rep.

[7] Yang J, Benyamin B, McEvoy BP, Gordon S, Henders AK, Nyholt DR, Madden PA, Heath AC, Martin NG, Montgomery GW, Goddard ME, Visscher PM (2010). Common SNPs explain a large proportion of the heritability for human height. Nat Genet.

[8] Tonjes A, Scholz M, Breitfeld J, Marzi C, Grallert H, Gross A, Ladenvall C, Schleinitz D, Krause K, Kirsten H, Laurila E, Kriebel J, Thorand B, Rathmann W, Groop L, Prokopenko I, Isomaa B, Beutner F, Kratzsch J, Thiery J, Fasshauer M, Kloting N, Gieger C, Bluher M, Stumvoll M, Kovacs P (2014). Genome wide meta-analysis highlights the role of genetic variation in RARRES2 in the regulation of circulating serum chemerin. PLoS Genet.

[9] Belonogova NM, Svishcheva GR, van Duijn CM, Aulchenko YS, Axenovich TI (2013). Region-based association analysis of human quantitative traits in related individuals. PLoS ONE.

[10] Teyssedre S, Elsen JM, Ricard A (2012). Statistical distributions of test statistics used for quantitative trait association mapping in structured populations. Genet Sel Evol.

[11] McArdle PF, O’Connell JR, Pollin TI, Baumgarten M, Shuldiner AR, Peyser PA, Mitchell BD (2007). Accounting for relatedness in family based genetic association studies. Hum Hered.

[12] Devlin B, Roeder K (1999). Genomic control for association studies. Biometrics.

[13] Gross A, Tonjes A, Kovacs P, Veeramah KR, Ahnert P, Roshyara NR, Gieger C, Rueckert IM, Loeffler M, Stoneking M, Wichmann HE, Novembre J, Stumvoll M, Scholz M (2011). Population-genetic comparison of the Sorbian isolate population in Germany with the German KORA population using genome-wide SNP arrays. BMC Genet.

[14] Aulchenko YS (2011). Chapter 9 - Effects of Population Structure in Genome-wide Association Studies. Analysis of Complex Disease Association Studies.

[15] Wang J (2002). An estimator for pairwise relatedness using molecular markers. Genetics.

[16] Stuart A, Ord K, Arnold S (1999). Kendall’s Advanced Theory of Statistics vol. 2A.

[17] Czado C, Schmidt T (2011). Mathematische Statistik. Statistik und ihre Anwendungen.

[18] HapMap. Merged phase I+II and III genotype files. ftp://ftp.ncbi.nlm.nih.gov/hapmap/genotypes/2010-08_phaseII+III/. Accessed 14 Mar 2017.

[19] Veeramah KR, Tonjes A, Kovacs P, Gross A, Wegmann D, Geary P, Gasperikova D, Klimes I, Scholz M, Novembre J, Stumvoll M (2011). Genetic variation in the Sorbs of eastern Germany in the context of broader European genetic diversity. Eur J Hum Genet.

[20] R Core Team. R: A Language and Environment for Statistical Computing. Vienna, Austria. https://www.R-project.org/. Accessed 14 Mar 2017.

[21] Astle W, Balding DJ (2009). Population Structure and Cryptic Relatedness in Genetic Association Studies. Statist Sci.

[22] Zhao K, Aranzana MJ, Kim S, Lister C, Shindo C, Tang C, Toomajian C, Zheng H, Dean C, Marjoram P, Nordborg M (2007). An Arabidopsis example of association mapping in structured samples. PLoS Genet.

[23] Bacanu SA, Devlin B, Roeder K (2002). Association studies for quantitative traits in structured populations. Genet Epidemiol.

[24] Price AL, Zaitlen NA, Reich D, Patterson N (2010). New approaches to population stratification in genome-wide association studies. Nat Rev Genet.

[25] Zhou H, Blangero J, Dyer TD, Chan KK, Lange K, Sobel EM (2017). Fast Genome-Wide QTL Association Mapping on Pedigree and Population Data. Genet Epidemiol.

[26] Zhou X, Stephens M (2012). Genome-wide efficient mixed-model analysis for association studies. Nat Genet.

[27] Yang J, Lee SH, Goddard ME, Visscher PM (2011). GCTA: a tool for genome-wide complex trait analysis. Am J Hum Genet.

[28] Yang J, Zaitlen NA, Goddard ME, Visscher PM, Price AL (2014). Advantages and pitfalls in the application of mixed-model association methods. Nat Genet.

[29] Eu-Ahsunthornwattana J, Miller EN, Fakiola M, Jeronimo SM, Blackwell JM, Cordell HJ (2014). Comparison of methods to account for relatedness in genome-wide association studies with family-based data. PLoS Genet.

[30] Maxima. A Computer Algebra System. Version 5.38.1. http://maxima.sourceforge.net/. Accessed 14 Mar 2017.

