# Supplementary material for: On the impact of relatedness on SNP association analysis
Source: BMC Genet. 2017 Dec 6;18:104. doi: 10.1186/s12863-017-0571-x (PMC5719591; doi:10.1186/s12863-017-0571-x)
Supplement: Supplementary file 2 — Theoretical background. This file provides the theoretical background and derivations of equations presented in the manuscript. (PDF 231 kb) [file 12863_2017_571_MOESM2_ESM.pdf]

Theoretical Background

October 20, 2017

Contents

1 Notation **2**

2 Modelling a SNP - phenotype association **3**
2.1 The mixed model 3
2.2 The linear model 4

3 Expected variance inflation **6**
3.1 Genotype probabilities 6
3.2 Estimation of variance inflation 7
3.3 Relationship between heritability and inflation 8
3.4 Example family structures 9

4 Empirical variances under relatedness **10**
4.1 Empirical variance of the SNP genotypes 10
4.2 Empirical variance of the effect estimate 10

5 Hypothesis testing **13**
5.1 Introduction 13
5.2 The null hypothesis 14
5.3 The alternative hypothesis 14

6 Genomic control **15**

7 List of symbols **17**

References **19**

1 Notation

First, we provide a short description of the major quantities used in the following. Vectors such as $\mathbf{s} = (s_1, s_2, ..., s_n)^{\mathrm{T}}$ consisting of n elements $s_1, s_2, ..., s_n$ and matrices as $\mathbf{G} = (G_{ij})$ composed of elements G_{ij} are printed in bold face. An element, e.g. of the product of matrices $\mathbf{M}$ and $\mathbf{F}$, is addressed by $(\mathbf{MF})_{ij}$. For the mean of $\mathbf{s}$ we use

$$\bar{s} = \frac{1}{n} \sum_{i=1}^{n} s_i$$

and for the mean of squared elements of $\mathbf{s}$ we write

$$\tilde{s} = \frac{1}{n} \sum_{i=1}^{n} s_i^2.$$

The sum of squared differences of $\mathbf{s}$ from its mean is expressed as

$$s_{\mathrm{q}} = \sum_{i=1}^{n} (s_i - \bar{s})^2 = \sum_{i=1}^{n} s_i^2 - \frac{1}{n} \left(\sum_{i=1}^{n} s_i \right)^2 .$$

Especially when used within a matrix, the limits of sums are omitted, e.g. $\sum s_i$ refers to a sum with index i ranging from 1 to n. This is also the case for nested sums as $\sum \sum s_{ij}$ where both indices i and j ranging from 1 to n. Occasionally, $j \neq i = 1$ is used as a lower limit for a sum, e.g. in $\sum_{i=1}^{n} \sum_{j \neq i=1}^{n} G_{ij}$. This means that the second index j ranges from 1 to n except for i, i.e. the diagonal of the matrix is omitted. Likewise, we abbreviate sums as

$$G_2 = \sum_{i=1}^{n} \sum_{j \neq i=1}^{n} G_{ij}^2$$

which is used for the sum of squared elements or

$$G_{\mathrm{r}} = \sum_{i=1}^{n} \left(\sum_{j \neq i=1}^{n} G_{ij} \right)^2$$

which is used for the sum of the squared row sums of $\mathbf{G}$ omitting the diagonal. The mean of these matrix elements is given by

$$\bar{G} = \frac{1}{n(n-1)} \sum_{i=1}^{n} \sum_{j \neq i=1}^{n} G_{ij}.$$

In Section 7, we present a comprehensive list of symbols along with their descriptions.

2 Modelling a SNP - phenotype association

2.1 The mixed model

According to [1, 2], an appropriate model for phenotypes $\mathbf{y} = (y_1, y_2, ..., y_n)^{\mathrm{T}}$ (n number of samples), which depends linearly on a SNP with genotypes $\mathbf{s} = (s_1, s_2, ..., s_n)^{\mathrm{T}}$ with intercept b_1 and slope b_2, is as follows

$$y_i = b_1 + b_2 s_i + g_i + e_i. \tag{1}$$

The polygenic effects $\mathbf{g} = (g_1, g_2, ..., g_n)^{\mathrm{T}}$ are modelled as random effects with a covariance matrix composed of σ_{g}^2 multiplied with the pairwise relatedness matrix $\mathbf{G} = (G_{ij})$

$$\mathbf{g} \sim \mathrm{N}_n(\mathbf{0}, \sigma_{\mathrm{g}}^2 \mathbf{G}). \tag{2}$$

For description of relatedness G_{ij}, we used the notation presented in [3]. A short introduction is given in Section 3.1. Residuals $\mathbf{e} = (e_1, e_2, ..., e_n)^{\mathrm{T}}$ are assumed to be independent normally distributed with variance σ_{e}^2

$$\mathbf{e} \sim \mathrm{N}_n(\mathbf{0}, \sigma_{\mathrm{e}}^2 \mathbf{I}). \tag{3}$$

The heritability of $\mathbf{y}$ can be expressed as

$$R_{\mathrm{h}}^2 = \frac{\sigma_{\mathrm{g}}^2}{\sigma_{\mathrm{g}}^2 + \sigma_{\mathrm{e}}^2}. \tag{4}$$

For given variance $\sigma_{\mathrm{e}}^2 > 0$ and assuming $R_{\mathrm{h}}^2 < 1$, the variance σ_{g}^2 can be derived

$$\sigma_{\mathrm{g}}^2 = \frac{\sigma_{\mathrm{e}}^2 R_{\mathrm{h}}^2}{1 - R_{\mathrm{h}}^2}.$$

Both random variables $\mathbf{g}$ and $\mathbf{e}$ of the mixed model in Eq. (1) can be combined to $\mathbf{f} = \mathbf{g} + \mathbf{e} = (f_1, f_2, ..., f_n)^{\mathrm{T}}$ so that

$$y_i = b_1 + b_2 s_i + f_i. \tag{5}$$

Given the expectation and variance of $\mathbf{g}$ in Eq. (2) and $\mathbf{e}$ in Eq. (3), it holds that

$$\mathbf{f} \sim \mathrm{N}_n(\mathbf{0}, \mathbf{F}) \tag{6}$$

where $\mathbf{F} = (F_{ij})$ and

$$F_{ij} = \begin{cases} \sigma_{\mathrm{g}}^2 + \sigma_{\mathrm{e}}^2 & \text{if } i = j \\ \sigma_{\mathrm{g}}^2 G_{ij} & \text{if } i \neq j. \end{cases} \tag{7}$$

Further, it holds that

$$\sigma_{\mathrm{g}}^2 + \sigma_{\mathrm{e}}^2 = \frac{\sigma_{\mathrm{e}}^2}{1 - R_{\mathrm{h}}^2}.$$

After factoring out $\sigma_{\mathrm{g}}^2 + \sigma_{\mathrm{e}}^2$ and replacement as shown, one obtains

$$F_{ij} = \frac{\sigma_{\mathrm{e}}^2}{1 - R_{\mathrm{h}}^2} \begin{cases} 1 & \text{if } i = j \\ R_{\mathrm{h}}^2 G_{ij} & \text{if } i \neq j. \end{cases} \tag{8}$$

Finally, we define

$$\mathbf{S} = \begin{pmatrix} 1 & 1 & \cdots & 1 \\ s_1 & s_2 & \cdots & s_n \end{pmatrix}^{\mathrm{T}}$$

and

$$\mathbf{b} = (b_1 \quad b_2)^{\mathrm{T}}.$$

Then, Eq. (5) can be rewritten as

$$\mathbf{y} = \mathbf{Sb} + \mathbf{f}. \tag{9}$$

2.2 The linear model

Ignoring relatedness, phenotypes $\mathbf{y}$ can be modelled by Eq. (9) and one can analyse the simple linear model

$$\mathbf{y} = \mathbf{S}\boldsymbol{\beta} + \boldsymbol{\epsilon}. \tag{10}$$

For beta coefficients $\boldsymbol{\beta}$ and independent normally distributed residuals $\boldsymbol{\epsilon}$, we assume

$$\boldsymbol{\beta} = (\beta_1 \quad \beta_2)^{\mathrm{T}}$$

and

$$\boldsymbol{\epsilon} \sim \mathrm{N}_n(\mathbf{0}, \sigma_\epsilon^2 \mathbf{I}).$$

According to [4], the least squares estimators for $\boldsymbol{\beta}$ are

$$\hat{\boldsymbol{\beta}} = (\mathbf{S}^{\mathrm{T}}\mathbf{S})^{-1}\mathbf{S}^{\mathrm{T}}\mathbf{y}. \tag{11}$$

As we are focussing on a SNP-phenotype association, we are interested in deriving the expected value and variance of the $\hat{\beta}_2$ estimator. Similar to [4], we calculate the expected values of the least squares estimators shown in Eq. (11) and phenotypes modelled by Eq. (9) as follows

$$\begin{aligned} \mathrm{E}(\hat{\boldsymbol{\beta}}) &= \mathrm{E}((\mathbf{S}^{\mathrm{T}}\mathbf{S})^{-1}\mathbf{S}^{\mathrm{T}}\mathbf{y}) \\ &= \mathrm{E}((\mathbf{S}^{\mathrm{T}}\mathbf{S})^{-1}\mathbf{S}^{\mathrm{T}}(\mathbf{Sb} + \mathbf{f})) \\ &= \mathbf{b} + (\mathbf{S}^{\mathrm{T}}\mathbf{S})^{-1}\mathbf{S}^{\mathrm{T}}\mathrm{E}(\mathbf{f}) = \mathbf{b} \end{aligned} \tag{12}$$

and obtain

$$\mathrm{E}(\hat{\beta}_2) = b_2. \tag{13}$$

Thus, there is no bias due to relatedness. In analogy to the calculations in [4], we use

$$\begin{aligned} \hat{\boldsymbol{\beta}} - \mathbf{b} &= (\mathbf{S}^{\mathrm{T}}\mathbf{S})^{-1}\mathbf{S}^{\mathrm{T}}(\mathbf{Sb} + \mathbf{f}) - \mathbf{b} \\ &= (\mathbf{S}^{\mathrm{T}}\mathbf{S})^{-1}\mathbf{S}^{\mathrm{T}}\mathbf{f} \end{aligned}$$

and the expected value of $\hat{\boldsymbol{\beta}}$ from Eq. (12) to obtain

$$\begin{aligned} \mathrm{V}(\hat{\boldsymbol{\beta}}) &= \mathrm{E}((\hat{\boldsymbol{\beta}}-\mathbf{b})(\hat{\boldsymbol{\beta}}-\mathbf{b})^{\mathrm{T}}) \\ &= (\mathbf{S}^{\mathrm{T}}\mathbf{S})^{-1}\mathbf{S}^{\mathrm{T}}\mathrm{E}(\mathbf{f}\mathbf{f}^{\mathrm{T}})\mathbf{S}(\mathbf{S}^{\mathrm{T}}\mathbf{S})^{-1} \\ &= (\mathbf{S}^{\mathrm{T}}\mathbf{S})^{-1}\mathbf{S}^{\mathrm{T}}\mathbf{F}\mathbf{S}(\mathbf{S}^{\mathrm{T}}\mathbf{S})^{-1}. \end{aligned} \tag{14}$$

By substitution of

$$\begin{aligned} (\mathbf{S}^{\mathrm{T}}\mathbf{S})^{-1} &= \begin{pmatrix} n & \sum s_i \\ \sum s_i & \sum s_i^2 \end{pmatrix}^{-1} \\ &= \frac{1}{n\sum_{i=1}^n s_i^2 - (\sum_{i=1}^n s_i)^2} \begin{pmatrix} \sum s_i^2 & -\sum s_i \\ -\sum s_i & n \end{pmatrix} \\ &= s_{\mathrm{q}}^{-1} \begin{pmatrix} \tilde{s} & -\bar{s} \\ -\bar{s} & 1 \end{pmatrix} \end{aligned} \tag{15}$$

in Eq. (14), we get

$$\mathrm{V}(\hat{\boldsymbol{\beta}}) = s_{\mathrm{q}}^{-2} \begin{pmatrix} \tilde{s} & -\bar{s} \\ -\bar{s} & 1 \end{pmatrix} \mathbf{S}^{\mathrm{T}}\mathbf{F}\mathbf{S} \begin{pmatrix} \tilde{s} & -\bar{s} \\ -\bar{s} & 1 \end{pmatrix}.$$

If we apply

$$\mathbf{S}^{\mathrm{T}}\mathbf{F}\mathbf{S} = \begin{pmatrix} \sum\sum F_{ij} & \sum\sum F_{ij}s_i \\ \sum\sum F_{ij}s_i & \sum\sum F_{ij}s_is_j \end{pmatrix}$$

and denote unneeded elements as “*” since we aim to derive $\mathrm{V}(\hat{\beta}_2)$ only, it holds that

$$\mathrm{V}(\hat{\boldsymbol{\beta}}) = s_{\mathrm{q}}^{-2} \begin{pmatrix} * & * \\ -\bar{s} & 1 \end{pmatrix} \begin{pmatrix} \sum\sum F_{ij} & \sum\sum F_{ij}s_i \\ \sum\sum F_{ij}s_i & \sum\sum F_{ij}s_is_j \end{pmatrix} \begin{pmatrix} * & -\bar{s} \\ * & 1 \end{pmatrix}.$$

Then, calculation of $\mathrm{V}(\hat{\beta}_2)$ yields

$$\mathrm{V}(\hat{\beta}_2) = s_{\mathrm{q}}^{-2} \sum_{i=1}^n \sum_{j=1}^n F_{ij}(\bar{s}^2 - 2\bar{s}s_i + s_is_j).$$

Replacing F_{ij} by Eq. (8) and factoring out elements with $i = j$ using

$$\sum_{i=1}^n \bar{s}^2 - 2\bar{s}s_i + s_i^2 = s_{\mathrm{q}}$$

we get

$$\mathrm{V}(\hat{\beta}_2) = s_{\mathrm{q}}^{-2} \frac{\sigma_{\mathrm{e}}^2}{1-R_{\mathrm{h}}^2} \left(s_{\mathrm{q}} + R_{\mathrm{h}}^2 \sum_{i=1}^n \sum_{j\neq i=1}^n G_{ij}(\bar{s}^2 - 2\bar{s}s_i + s_is_j) \right).$$

Re-substituting s_{q} by the term presented in Section 1 yields

$$\mathrm{V}(\hat{\beta}_2) = \frac{\sigma_{\mathrm{e}}^2}{\sum_{i=1}^n (s_i-\bar{s})^2} \frac{1}{1-R_{\mathrm{h}}^2} \left(1 + R_{\mathrm{h}}^2 \frac{\sum_{i=1}^n \sum_{j\neq i=1}^n G_{ij}(\bar{s}^2 - 2\bar{s}s_i + s_is_j)}{\sum_{i=1}^n (s_i-\bar{s})^2} \right).$$

Without heritability, i.e. $R_\mathrm{h}^2 = 0$, relatedness has no impact and the variance of the beta estimator simplifies to

$$V_\beta = \frac{\sigma_\mathrm{e}^2}{\sum_{i=1}^{n}(s_i - \bar{s})^2} \tag{16}$$

corresponding to the standard linear model as described in [4]. We define

$$\lambda = 1 + R_\mathrm{h}^2 \frac{\sum_{i=1}^{n}\sum_{j\neq i=1}^{n} G_{ij}(\bar{s}^2 - 2\bar{s}s_i + s_i s_j)}{\sum_{i=1}^{n}(s_i - \bar{s})^2}. \tag{17}$$

Then, the inflation of $\mathrm{V}(\hat{\beta}_2)$ with respect to V_β is $\lambda/(1-R_\mathrm{h}^2)$ and the variance of $\hat{\beta}_2$ can be rewritten as

$$\mathrm{V}(\hat{\beta}_2) = \frac{\lambda}{1 - R_\mathrm{h}^2} V_\beta. \tag{18}$$

As we will see in Section 4.2, the empirical variance of the beta estimate is also inflated by factor $1/(1-R_\mathrm{h}^2)$. Hence, this factor is cancelled out when estimating the corresponding T statistic.

3 Expected variance inflation

In contrast to Eq. (17) where concrete genotypes are required to calculate λ, we calculate below the expected inflation given the relatedness matrix.

3.1 Genotype probabilities

We assume that only two different SNP alleles at the same locus of individual i and individual j are present. Following [3], we denote ϕ_{ij} and δ_{ij} as the probabilities that exactly one allele, respectively both alleles, are inherited from a common ancestor (identical by descent). Then, relatedness is defined as

$$G_{ij} = \phi_{ij}/2 + \delta_{ij}.$$

We denote the allele frequency of an arbitrary reference allele as p and the allele frequency of the other allele as $q = 1 - p$. Genotypes s_i and s_j belong to individuals i and j, respectively. Each genotype consists of 0, 1 or 2 references alleles. According to [3], for a pair of genotypes s_i, s_j it holds

$$\begin{aligned}
\mathrm{P}(s_i = 1, s_j = 1) &= 4p^2q^2 + pq(1-4pq)\phi_{ij} + 2pq(1-2pq)\delta_{ij} \\
\mathrm{P}(s_i = 1, s_j = 2) &= 4pq^3 + 2pq^2(1-2q)\phi_{ij} - 4pq^3\delta_{ij} \\
\mathrm{P}(s_i = 2, s_j = 2) &= q^4 + pq^3\phi_{ij} + q^2(1-q^2)\delta_{ij}.
\end{aligned}$$

For the expected value of $s_i s_j$ we obtain

$$\begin{aligned}
\mathrm{E}(s_i s_j) &= 1\mathrm{P}(s_i = 1, s_j = 1) + 2\mathrm{P}(s_i = 1, s_j = 2) + 4\mathrm{P}(s_i = 2, s_j = 2) \\
&= 4q^2 + pq\phi_{ij} + 2pq\delta_{ij} \\
&= 4q^2 + 2pqG_{ij}.
\end{aligned} \tag{19}$$

Accordingly, for the expected value of s_i^2 it follows

$$\mathrm{E}(s_i^2) = 4q^2 + 2pq. \tag{20}$$

3.2 Estimation of variance inflation

Using Taylor expansion, the expectation of the quotient of two random variables X_1 and X_2 can be estimated by

$$\mathrm{E}\left(\frac{X_1}{X_2}\right) \approx \frac{\mathrm{E}(X_1)}{\mathrm{E}(X_2)} - \frac{\mathrm{Cov}(X_1, X_2)}{\mathrm{E}(X_2)^2} + \frac{\mathrm{E}(X_1)}{\mathrm{E}(X_2)^3}\mathrm{V}(X_2).$$

If $\mathrm{Cov}(X_1, X_2)$ and $\mathrm{V}(X_2)$ are small compared to $\mathrm{E}(X_2)$, then it approximately holds that

$$\mathrm{E}\left(\frac{X_1}{X_2}\right) \approx \frac{\mathrm{E}(X_1)}{\mathrm{E}(X_2)}.$$

Based on this approximation, we estimate $\mathrm{E}(\bar{s}^2 - 2\bar{s}s_i + s_i s_j)$ and $\mathrm{E}\left(\sum_{i=1}^n (s_i - \bar{s})^2\right)$ of Eq. (17) in order to derive the expected variance inflation. Applying Eq. (19) and Eq. (20) one obtains

$$\begin{aligned}
\mathrm{E}(\bar{s}^2 - 2\bar{s}s_i + s_i s_j) &= \mathrm{E}\left(\frac{1}{n^2}\sum_{k=1}^n\sum_{l=1}^n s_k s_l - \frac{2}{n}\sum_{k=1}^n s_k s_i + s_i s_j\right) \\
&= \frac{1}{n^2}\left(4q^2n^2 + 2pqn + 2pq\sum_{k=1}^n\sum_{l\neq k=1}^n G_{kl}\right) - \\
&\quad \frac{2}{n}\left(4q^2n + 2pq + 2pq\sum_{k\neq i=1}^n G_{ki}\right) + \\
&\quad 4q^2 + 2pqG_{ij} \\
&= 2pq\left(G_{ij} + \frac{1}{n^2}\sum_{k=1}^n\sum_{l\neq k=1}^n G_{kl} - \frac{2}{n}\sum_{k\neq i=1}^n G_{ki} - \frac{1}{n}\right)
\end{aligned} \tag{21}$$

and

$$\begin{aligned}
\mathrm{E}\left(\sum_{i=1}^n (s_i - \bar{s})^2\right) &= \mathrm{E}\left(\sum_{i=1}^n s_i^2 - \frac{1}{n}\sum_{i=1}^n\sum_{j=1}^n s_i s_j\right) \\
&= n(4q^2 + 2pq) - \frac{1}{n}\left(4q^2n^2 + 2pqn + 2pq\sum_{i=1}^n\sum_{j\neq i=1}^n G_{ij}\right) \\
&= 2pq\left(n - 1 - \frac{1}{n}\sum_{i=1}^n\sum_{j\neq i=1}^n G_{ij}\right).
\end{aligned} \tag{22}$$

If $\mathrm{Cov}(\sum_{i=1}^n \sum_{j \neq i=1}^n G_{ij}(\bar{s}^2 - 2\bar{s}s_i + s_i s_j), \sum_{i=1}^n (s_i - \bar{s})^2)$ and $\mathrm{V}(\sum_{i=1}^n (s_i - \bar{s})^2)$ of Eq. (17) are small compared to $\mathrm{E}\left(\sum_{i=1}^n (s_i - \bar{s})^2\right)$, the expected value of λ can be approximated by Eq. (21) and Eq. (22) as follows

$$\begin{aligned}
\mathrm{E}(\lambda) &\approx 1 + R_{\mathrm{h}}^2 \frac{\sum_{i=1}^n \sum_{j \neq i=1}^n G_{ij} \mathrm{E}(\bar{s}^2 - 2\bar{s}s_i + s_i s_j)}{\mathrm{E}\left(\sum_{i=1}^n (s_i - \bar{s})^2\right)} \\
&= 1 + R_{\mathrm{h}}^2 \frac{\sum_{i=1}^n \sum_{j \neq i=1}^n G_{ij} \left(G_{ij} - \frac{2}{n} \sum_{k \neq i=1}^n G_{ki} + \frac{1}{n^2} \sum_{k=1}^n \sum_{l \neq k=1}^n G_{kl} - \frac{1}{n} \right)}{n - 1 - \frac{1}{n} \sum_{i=1}^n \sum_{j \neq i=1}^n G_{ij}}.
\end{aligned} \tag{23}$$

Interestingly, this term is independent of the allele frequency p. By expanding the numerator and introducing the mean relatedness $\bar{G}$, the sum of squared elements G_2 and the sum of squared row sums G_{r} as described in Section 1, we obtain

$$\begin{aligned}
\mathrm{E}(\lambda) &\approx 1 + R_{\mathrm{h}}^2 \frac{\sum_{i=1}^n \sum_{j \neq i=1}^n G_{ij}^2 - \frac{2}{n} \sum_{i=1}^n \left(\sum_{j \neq i=1}^n G_{ij} \right)^2 + (n-1)^2 \bar{G}^2 - (n-1)\bar{G}}{(n-1)(1-\bar{G})} \\
&= 1 + R_{\mathrm{h}}^2 \left(\frac{\sum_{i=1}^n \sum_{j \neq i=1}^n G_{ij}^2 - \frac{2}{n} \sum_{i=1}^n \left(\sum_{j \neq i=1}^n G_{ij} \right)^2}{(n-1)(1-\bar{G})} + \frac{(n-1)\bar{G}^2 - \bar{G}}{1-\bar{G}} \right) \\
&= 1 + R_{\mathrm{h}}^2 \left(\frac{G_2 - \frac{2}{n} G_{\mathrm{r}}}{(n-1)(1-\bar{G})} + \frac{(n-1)\bar{G}^2 - \bar{G}}{1-\bar{G}} \right).
\end{aligned}$$

Additionally, for small mean relatedness $\bar{G} \approx 0$ and

$$\lambda' = 1 + R_{\mathrm{h}}^2 \frac{G_2 - \frac{2}{n} G_{\mathrm{r}}}{n-1} \tag{24}$$

it holds that $\mathrm{E}(\lambda) \approx \lambda'$.

3.3 Relationship between heritability and inflation

Assume that a certain inflation λ' and a heritability R_{h}^2 are given. Then, one could ask what heritability R_{t}^2 is needed so that λ' changes to λ'_{t}. Conversely, one could be interested in what λ'_{t} results from assuming R_{t}^2 instead of R_{h}^2. Both questions are answered as follows. After substituting

$$G_{\mathrm{s}} = \frac{G_2 - \frac{2}{n} G_{\mathrm{r}}}{n-1}$$

in Eq. (24), we obtain

$$\lambda' = 1 + R_{\mathrm{h}}^2 G_{\mathrm{s}}.$$

Solving for G_{s}, it is

$$G_{\mathrm{s}} = \frac{\lambda' - 1}{R_{\mathrm{h}}^2}.$$

Similarly, for λ'_{t} it holds that

$$G_{\mathrm{s}} = \frac{\lambda'_{\mathrm{t}} - 1}{R^2_{\mathrm{t}}}.$$

Equalising the latter two equations results in a relation of inflation and heritability for a given relatedness structure

$$(\lambda' - 1)R^2_{\mathrm{t}} = (\lambda'_{\mathrm{t}} - 1)R^2_{\mathrm{h}}.$$

Based on this equation, it follows simply by transformation

$$\begin{aligned} R^2_{\mathrm{t}} &= \frac{\lambda'_{\mathrm{t}} - 1}{\lambda' - 1} R^2_{\mathrm{h}}, \\ \lambda'_{\mathrm{t}} &= 1 + (\lambda' - 1)\frac{R^2_{\mathrm{t}}}{R^2_{\mathrm{h}}}. \end{aligned}$$

3.4 Example family structures

Inflation λ' in Eq. (24) can be estimated for arbitrary family structures. For illustration, we consider a study with f families with one father per family. Each father is mated with m mothers and each mother has c children. If only siblings are considered for the study then the number of study samples is $n = cmf$. For this family structure, one obtains

$$\begin{aligned} G_2 &= \left(\frac{1}{4}c(c-1) + \frac{1}{16}c^2(m-1)\right) mf, \\ G_{\mathrm{r}} &= \left(\frac{1}{2}(c-1) + \frac{1}{4}c(m-1)\right)^2 cmf \end{aligned}$$

Using the software MAXIMA [5], these terms can be used to calculate $\lambda'_{f;m;c}$ as given in Eq. (24). It holds that

$$\lambda'_{f;m;c} = 1 + R^2_{\mathrm{h}} \frac{(c^2 f - 2c^2)m^2 + ((3c^2 - 4c)f - 4c^2 + 8c)m - 2c^2 + 8c - 8}{16cmf - 16}.$$

The case is more complicate when all individuals, i.e. offspring and parents, are considered. Then the number of individuals is $n = (cm + m + 1)f$ and we get

$$\begin{aligned} G_2 &= \left(\frac{1}{4}c(c-1) + \frac{1}{16}c^2(m-1) + c\right) mf \\ G_{\mathrm{r}} &= \left(\frac{1}{2}(c-1) + \frac{1}{4}c(m-1) + 1\right)^2 cmf + \left(\frac{1}{2}mc\right)^2 f + \left(\frac{1}{2}c\right)^2 mf. \end{aligned}$$

Using MAXIMA again for calculation of $\lambda'_{f;m;c}$ as presented in Eq. (24), we obtain

$$\begin{aligned} \lambda'_{f;m;c} = 1 + R^2_{\mathrm{h}}[&((c^3 + c^2)f - 2c^3)m^3 + ((3c^3 + 16c^2 + 12c)f - 4c^3 - 16c^2)m^2 + \\ &((3c^2 + 12c)f - 2c^3 - 16c^2 - 8c)m]/[(16c^2 + 32c + 16)fm^2 + \\ &((32c + 32)f - 16c - 16)m + 16f - 16]. \end{aligned} \quad (25)$$

For reproducing the results, the Maxima document which was used for derivation of the formulae is provided as supplemental material. A study consisting of f trio families ($m = 1$, $c = 1$) is an example for Eq. (25). In this special case the number of individuals is $n = 3f$ and the expected variance inflation is

$$\lambda'_{f;1;1} = 1 + R_\mathrm{h}^2 \frac{f-1}{3f-1}.$$

Thus, different degrees of variance inflation can be expressed through R_h^2. For example, under maximal heritability $R_\mathrm{h}^2 = 1$, $\lambda'_{f;1;1}$ converges to 4/3 with increasing f. Stronger inflation can be achieved by an increased number of relationships, e.g. by assuming $m = 2$, $c = 3$ (i.e. $n = 9f$) with

$$\lambda'_{f;2;3} = 1 + R_\mathrm{h}^2 \frac{243f - 314}{216f - 24}.$$

Under maximal heritability $R_\mathrm{h}^2 = 1$, $\lambda'_{f;2;3}$ converges to 17/8 with increasing f.

4 Empirical variances under relatedness

4.1 Empirical variance of the SNP genotypes

Referring to the calculation of $\mathrm{E}(s_\mathrm{q})$ in Eq. (22), the expected value of the empirical SNP variance S_s^2 for a SNP with n genotypes and allele frequency p ($q = 1 - p$) reads as follows

$$\mathrm{E}(S_\mathrm{s}^2) = \mathrm{E}(\frac{s_\mathrm{q}}{n-1}) = 2pq\left(1 - \frac{1}{n(n-1)}\sum_{i=1}^{n}\sum_{j\neq i=1}^{n} G_{ij}\right) = 2pq(1-\bar{G}).$$

As long as $\bar{G}$ is close to zero, relatedness causes only a small negative bias and we obtain $\mathrm{E}(S_\mathrm{s}^2) \approx 2pq$ which corresponds to the expected genotype variance.

4.2 Empirical variance of the effect estimate

Without heritability, the variance of $\hat{\beta}_2$ is given in Eq. (16), but when testing for a phenotype-SNP association, the residual variance σ_ϵ^2 needs to be determined. It can be estimated by S_ϵ^2 [4] as follows

$$S_\epsilon^2 = \frac{(\mathbf{y} - \mathbf{S}\hat{\boldsymbol{\beta}})^\mathrm{T}(\mathbf{y} - \mathbf{S}\hat{\boldsymbol{\beta}})}{n-2}. \tag{26}$$

Further, the empirical variance of $\hat{\beta}_2$ is estimated by

$$S_\upbeta^2 = \frac{S_\epsilon^2}{\sum_{i=1}^{n}(s_i - \bar{s})^2} \tag{27}$$

given a linear model with two parameters (intercept and slope). According to [4], we derive the expectation of $S_\upbeta^2$ below. Using $\hat{\boldsymbol{\beta}}$ from Eq. (11) and $\mathbf{y}$ from Eq. (9), it is

$$\mathbf{y} - \mathbf{S}\hat{\boldsymbol{\beta}} = (\mathbf{I} - \mathbf{S}(\mathbf{S}^\mathrm{T}\mathbf{S})^{-1}\mathbf{S}^\mathrm{T})\mathbf{y} = (\mathbf{I} - \mathbf{S}(\mathbf{S}^\mathrm{T}\mathbf{S})^{-1}\mathbf{S}^\mathrm{T})\mathbf{f} = \mathbf{M}\mathbf{f}$$

where

$$\mathbf{M} = \mathbf{I} - \mathbf{S}(\mathbf{S}^\mathrm{T}\mathbf{S})^{-1}\mathbf{S}^\mathrm{T} = (M_{ij}). \tag{28}$$

As $\mathbf{M}$ is idempotent ($\mathbf{M}^2 = \mathbf{M} = \mathbf{M}^\mathrm{T}$), it follows

$$(\mathbf{y} - \mathbf{S}\hat{\boldsymbol{\beta}})^\mathrm{T}(\mathbf{y} - \mathbf{S}\hat{\boldsymbol{\beta}}) = \mathbf{f}^\mathrm{T}\mathbf{M}^\mathrm{T}\mathbf{M}\mathbf{f} = \mathbf{f}^\mathrm{T}\mathbf{M}\mathbf{f}.$$

Using the trace and its properties, we obtain

$$\mathrm{E}((\mathbf{y} - \mathbf{S}\hat{\boldsymbol{\beta}})^\mathrm{T}(\mathbf{y} - \mathbf{S}\hat{\boldsymbol{\beta}})) = \mathrm{E}(\mathbf{f}^\mathrm{T}\mathbf{M}\mathbf{f}) = \mathrm{E}(\mathrm{tr}(\mathbf{M}\mathbf{f}\mathbf{f}^\mathrm{T})) = \mathrm{tr}(\mathbf{M}\mathrm{E}(\mathbf{f}\mathbf{f}^\mathrm{T})) = \mathrm{tr}(\mathbf{M}\mathbf{F}). \tag{29}$$

Replacing $(\mathbf{S}^\mathrm{T}\mathbf{S})^{-1}$ in Eq. (28) by Eq. (15) it follows

$$\mathbf{M} = \mathbf{I} - s_\mathrm{q}^{-1}\mathbf{S}\begin{pmatrix} \tilde{s} & -\bar{s} \\ -\bar{s} & 1 \end{pmatrix}\mathbf{S}^\mathrm{T}.$$

Each element M_{ij} of $\mathbf{M}$ can be calculated by multiplication with $\mathbf{S}$ and introducing the Kronecker delta $\delta_{ij} = (i = j)$:

$$M_{ij} = \delta_{ij} - s_\mathrm{q}^{-1}(\tilde{s} - \bar{s}(s_i + s_j) + s_i s_j).$$

Further, multiplication of $\mathbf{M}$ with $\mathbf{F}$ yields the following elements

$$(\mathbf{M}\mathbf{F})_{ij} = \sum_{k=1}^{n} F_{kj}(\delta_{ik} - s_\mathrm{q}^{-1}(\tilde{s} - \bar{s}(s_i + s_k) + s_i s_k)).$$

For the trace of $\mathbf{M}\mathbf{F}$, we obtain

$$\mathrm{tr}(\mathbf{M}\mathbf{F}) = \sum_{i=1}^{n}(\mathbf{M}\mathbf{F})_{ii} = \sum_{i=1}^{n}\sum_{j=1}^{n} F_{ij}(\delta_{ij} - s_\mathrm{q}^{-1}(\tilde{s} - \bar{s}(s_i + s_j) + s_i s_j)).$$

After replacing $\mathbf{F}$ by Eq. (8), treating elements with $i = j$ separately and substituting δ_{ij} it follows

$$\begin{aligned} \mathrm{tr}(\mathbf{M}\mathbf{F}) \; = \; & \frac{\sigma_\mathrm{e}^2}{1 - R_\mathrm{h}^2}\sum_{i=1}^{n}(1 - s_\mathrm{q}^{-1}(\tilde{s} - 2\bar{s}s_i + s_i^2)) - \\ & \frac{\sigma_\mathrm{e}^2}{1 - R_\mathrm{h}^2} s_\mathrm{q}^{-1} R_\mathrm{h}^2 \sum_{i=1}^{n}\sum_{j \neq i = 1}^{n} G_{ij}(\tilde{s} - \bar{s}(s_i + s_j) + s_i s_j). \end{aligned}$$

The first sum can be simplified by re-substituting s_q, $\tilde{s}$ and $\bar{s}$ as defined in Section 1:

$$\begin{aligned}\sum_{i=1}^{n}\left(1 - s_\mathrm{q}^{-1}(\tilde{s} - 2\bar{s}s_i + s_i^2)\right) &= n - \frac{\sum_{i=1}^{n} s_i^2 - \frac{2}{n}\left(\sum_{i=1}^{n} s_i\right)^2 + \sum_{i=1}^{n} s_i^2}{\sum_{i=1}^{n}(s_i - \bar{s})^2} \\ &= n - \frac{2\left(\sum_{i=1}^{n} s_i^2 - \frac{1}{n}\left(\sum_{i=1}^{n} s_i\right)^2\right)}{\sum_{i=1}^{n}(s_i - \bar{s})^2} \\ &= n - 2.\end{aligned}$$

For the trace of $\mathbf{MF}$ it follows

$$\mathrm{tr}(\mathbf{MF}) = \frac{\sigma_\mathrm{e}^2}{1 - R_\mathrm{h}^2}\left(n - 2 - R_\mathrm{h}^2 \frac{\sum_{i=1}^{n}\sum_{j\neq i=1}^{n} G_{ij}(\tilde{s} - \bar{s}(s_i + s_j) + s_i s_j)}{s_\mathrm{q}}\right).$$

Finally, the expected value of $S_\upbeta^2$ can be calculated by using Eq. (27), Eq. (26), Eq. (29) and Eq. (16) as follows

$$\begin{aligned}\mathrm{E}(S_\upbeta^2) &= \frac{\mathrm{E}(S_\epsilon^2)}{\sum_{i=1}^{n}(s_i - \bar{s})^2} = \frac{\mathrm{E}((\mathbf{y} - \mathbf{S}\hat{\boldsymbol{\beta}})^\mathrm{T}(\mathbf{y} - \mathbf{S}\hat{\boldsymbol{\beta}}))}{(n-2)\sum_{i=1}^{n}(s_i - \bar{s})^2} = \frac{\mathrm{tr}(\mathbf{MF})}{(n-2)\sum_{i=1}^{n}(s_i - \bar{s})^2} \\ &= \frac{\sigma_\mathrm{e}^2}{(1 - R_\mathrm{h}^2)\sum_{i=1}^{n}(s_i - \bar{s})^2}\left(1 - R_\mathrm{h}^2 \frac{\sum_{i=1}^{n}\sum_{j\neq i=1}^{n} G_{ij}(\tilde{s} - \bar{s}(s_i + s_j) + s_i s_j)}{(n-2)\sum_{i=1}^{n}(s_i - \bar{s})^2}\right) \\ &= \frac{V_\upbeta}{1 - R_\mathrm{h}^2}\left(1 - R_\mathrm{h}^2 \frac{\sum_{i=1}^{n}\sum_{j\neq i=1}^{n} G_{ij}(\tilde{s} - \bar{s}(s_i + s_j) + s_i s_j)}{(n-2)\sum_{i=1}^{n}(s_i - \bar{s})^2}\right) \\ &= \frac{V_\upbeta}{1 - R_\mathrm{h}^2}\nu.\end{aligned}$$

This implies that $S_\upbeta^2$ is biased compared to $V_\upbeta/(1 - R_\mathrm{h}^2)$. It is interesting to investigate under which conditions the ν term is close to 1. Similar to the derivation of λ' as summarised in Eq. (23), we estimate $\mathrm{E}(\nu)$ by calculation of $\mathrm{E}(\tilde{s} - \bar{s}(s_i + s_j) + s_i s_j) = \mathrm{E}(\tilde{s} - 2\bar{s}s_i + s_i s_j)$ and $\mathrm{E}\left(\sum_{i=1}^{n}(s_i - \bar{s})^2\right)$. The difference is that $\tilde{s}$ is used in $\mathrm{E}(\tilde{s} - 2\bar{s}s_i + s_i s_j)$ in contrast to $\bar{s}^2$ in $\mathrm{E}(\bar{s}^2 - 2\bar{s}s_i + s_i s_j)$ as shown in Eq. (21). It holds that

$$\begin{aligned}\mathrm{E}(\tilde{s} - 2\bar{s}s_i + s_i s_j) &= \mathrm{E}\left(\frac{1}{n}\sum_{k=1}^{n} s_k^2 - \frac{2}{n}\sum_{k=1}^{n} s_k s_i + s_i s_j\right) \\ &= 4q^2 + 2pq - \\ &\quad \frac{2}{n}\left(4q^2 n + 2pq + 2pq\sum_{k\neq i=1}^{n} G_{ki}\right) + \\ &\quad 4q^2 + 2pqG_{ij} \\ &= 2pq\left(G_{ij} - \frac{2}{n}\sum_{k\neq i=1}^{n} G_{ki} + 1 - \frac{2}{n}\right).\end{aligned}$$

Utilizing this estimate and the estimate for $\mathrm{E}\left(\sum_{i=1}^{n}(s_i-\bar{s})^2\right)$ from Eq. (22), it holds

$$\begin{aligned}\mathrm{E}(\nu) &\approx 1-R_{\mathrm{h}}^2\frac{\sum_{i=1}^{n}\sum_{j\neq i=1}^{n}G_{ij}\mathrm{E}(\tilde{s}-\bar{s}(s_i+s_j)+s_is_j)}{(n-2)\mathrm{E}(\sum_{i=1}^{n}(s_i-\bar{s})^2)}\\ &= 1-R_{\mathrm{h}}^2\frac{\sum_{i=1}^{n}\sum_{j\neq i=1}^{n}G_{ij}\left(G_{ij}-\frac{2}{n}\sum_{k\neq i=1}^{n}G_{ki}+1-\frac{2}{n}\right)}{(n-2)\left(n-1-\frac{1}{n}\sum_{i=1}^{n}\sum_{j\neq i=1}^{n}G_{ij}\right)}.\end{aligned}$$

Expanding the enumerator and introducing the mean relatedness $\bar{G}$, the sum of squared elements G_2 and the sum of squared row sums G_{r} as described in Section 1, we obtain

$$\begin{aligned}\mathrm{E}(\nu) &\approx 1-R_{\mathrm{h}}^2\frac{\sum_{i=1}^{n}\sum_{j\neq i=1}^{n}G_{ij}^2-\frac{2}{n}\sum_{i=1}^{n}\left(\sum_{j\neq i=1}^{n}G_{ij}\right)^2+(n-1)(n-2)\bar{G}}{(n-1)(n-2)(1-\bar{G})}\\ &= 1-R_{\mathrm{h}}^2\left(\frac{G_2-\frac{2}{n}G_{\mathrm{r}}}{(n-1)(n-2)(1-\bar{G})}+\frac{\bar{G}}{1-\bar{G}}\right).\end{aligned}\tag{30}$$

The bias is zero, i.e. $\mathrm{E}(\nu)=1$, in the case of unrelatedness. The term in parenthesis is close to zero for well-behaved relatedness matrices as shown for our example studies. In this case, $\mathrm{E}(\nu)\approx 1$ and

$$\mathrm{E}(S_{\beta}^2)\approx\frac{V_{\beta}}{1-R_{\mathrm{h}}^2}.\tag{31}$$

5 Hypothesis testing

5.1 Introduction

Assume we observe phenotypes $\mathbf{y}$ and SNP genotypes $\mathbf{s}$ as modelled in Eq. (9) and we would like to know, whether the phenotype is associated with the SNP or not. This is done by using the simple model in Eq. (10) and testing against the null hypothesis of $\beta_2=0$ of no association. Precisely, the test statistic

$$T=\frac{\hat{\beta}_2}{S_{\beta}}$$

as presented in [6] is evaluated. The distribution properties of the T statistic under the null hypothesis are required for assessing the type I error. In contrast, the distribution properties under the alternative hypothesis are needed for calculating the power of the test. Thus, we assess below the distribution of T and their properties given these two premises. Since the random effects $\mathbf{f}$ in Eq. (6) are multivariate normally distributed, it follows that each component f_i of $\mathbf{f}$ is normally distributed. Further, each component $b_1+b_2s_i+f_i$ of $\mathbf{y}$ in Eq. (9) is normally distributed as the constants $b_1+b_2s_i$ do not change the class of distribution. Therefore, $\mathbf{y}$ is multivariate normal. The least squares estimators $\hat{\boldsymbol{\beta}}$ in Eq. (11) are a linear transformation of $\mathbf{y}$ and thus multivariate normal [6], too. As the effect estimate $\hat{\beta}_2$ is a component of $\hat{\boldsymbol{\beta}}$, it is normally distributed. If the

variance of S_β^2 is small, one can replace S_β^2 by the expected value $\mathrm{E}(S_\beta^2)$. It follows that T is approximately normal and its expectation and variance can be assessed as follows

$$\begin{aligned} \mathrm{E}(T) &= \mathrm{E}\left(\frac{\hat{\beta}_2}{S_\beta}\right) \approx \frac{\mathrm{E}(\hat{\beta}_2)}{\sqrt{\mathrm{E}(S_\beta^2)}}, \\ \mathrm{V}(T) &= \mathrm{V}\left(\frac{\hat{\beta}_2}{S_\beta}\right) \approx \frac{\mathrm{V}(\hat{\beta}_2)}{\mathrm{E}(S_\beta^2)}. \end{aligned}$$

5.2 The null hypothesis

On the basis of these two approximations, the properties of T can be analysed under the null and alternative hypothesis. Under the null hypothesis, we assume $b_2 = 0$. Since $\mathrm{E}(\hat{\beta}_2) = b_2$ in Eq. (13) holds for both hypotheses, it follows that

$$\mathrm{E}(T) \approx \frac{\mathrm{E}(\hat{\beta}_2)}{\sqrt{\mathrm{E}(S_\beta^2)}} = \frac{b_2}{\sqrt{\mathrm{E}(S_\beta^2)}} = 0.$$

Further, using the inflation of $\mathrm{V}(\hat{\beta}_2)$ by $\lambda/(1 - R_\mathrm{h}^2)$ with respect to V_β as shown in Eq. (18) and the approximation for $\mathrm{E}(S_\beta^2)$ in Eq. (31), we obtain

$$\mathrm{V}(T) \approx \frac{\mathrm{V}(\hat{\beta}_2)}{\mathrm{E}(S_\beta^2)} \approx \frac{\frac{\lambda}{1-R_\mathrm{h}^2} V_\beta}{\frac{V_\beta}{1-R_\mathrm{h}^2}} = \lambda. \tag{32}$$

In summary, the distribution of T is approximately

$$T \sim \mathrm{N}(0, \lambda). \tag{33}$$

5.3 The alternative hypothesis

When comparing different SNPs, it is reasonable to assume a fixed explained variance by the SNP instead of a fixed SNP effect b_2 regarding Eq. (5). As the SNP variance depends on the allele frequency, a fixed b_2 would otherwise lead to varying contributions to the phenotype. For given σ_e^2, empirical SNP variance S_s^2, heritability R_h^2 as defined in Eq. (4), explained variance by the SNP

$$R_\mathrm{s}^2 = \frac{b_2^2 S_\mathrm{s}^2}{\sigma_\mathrm{g}^2 + \sigma_\mathrm{e}^2}$$

and $b_2 > 0$, we obtain

$$b_2 = \sqrt{\frac{R_\mathrm{s}^2 \sigma_\mathrm{e}^2}{S_\mathrm{s}^2 (1 - R_\mathrm{h}^2)}}. \tag{34}$$

The empirical variance S_s^2 of the SNP can be assessed as follows

$$S_\mathrm{s}^2 = \frac{s_\mathrm{q}}{n-1}.$$

As shown in Section 4.1, this estimate is biased due to relatedness. However, it has only subtle impact as long as the mean relatedness is low. Using the expectation of $\hat{\beta}_2$ from Eq. (13), the approximation for $\mathrm{E}(S_\beta^2)$ from Eq. (31), the assumption from Eq. (34) and the definition of V_β from Eq. (16), we obtain

$$\mathrm{E}(T) \approx \frac{\mathrm{E}(\hat{\beta}_2)}{\sqrt{\mathrm{E}(S_\beta^2)}} \approx \frac{b_2}{\sqrt{\frac{V_\beta}{1-R_\mathrm{h}^2}}} = \sqrt{(n-1)R_\mathrm{s}^2} = \mu. \tag{35}$$

Derivation of the variance is equivalent to Eq. (32) and yields $\mathrm{V}(T) \approx \lambda$. Therefore, it approximately holds that

$$T \sim \mathrm{N}(\mu, \lambda). \tag{36}$$

6 Genomic control

Genomic control as described in [7] is a well-established method for correcting the variance inflation of a test statistic. Below we provide a short introduction regarding the median of T statistics under the null hypothesis. Given a random variable $X \sim \mathrm{N}(0, \sigma^2)$ with probability density f_X, we are aiming at deriving the median of $|X|$. The probability distribution of $|X|$ is

$$\begin{aligned}
\mathrm{F}_{|X|}(x) &= \mathrm{P}(|X| < x) \\
&= \int_{-x}^{x} \mathrm{f}_X(y)\,\mathrm{d}y \\
&= 2\int_{0}^{x} \mathrm{f}_X(y)\,\mathrm{d}y \\
&= 2\int_{0}^{x} \frac{1}{\sqrt{2\pi\sigma^2}} \exp\left(-\frac{y^2}{2\sigma^2}\right)\mathrm{d}y.
\end{aligned}$$

Using the substitution $z = y/\sqrt{2\sigma^2}$, we obtain

$$\begin{aligned}
\mathrm{F}_{|X|}(x) &= 2\int_{0}^{x/\sqrt{2\sigma^2}} \frac{1}{\sqrt{2\pi\sigma^2}} \exp(-z^2)\sqrt{2\sigma^2}\,\mathrm{d}z \\
&= \frac{2}{\sqrt{\pi}} \int_{0}^{x/\sqrt{2\sigma^2}} \exp(-z^2)\,\mathrm{d}z \\
&= \mathrm{erf}(x/\sqrt{2\sigma^2})
\end{aligned}$$

with error function erf. For the median of $|X|$ it holds that $\mathrm{erf}(\mathrm{median}(|X|)/\sqrt{2\sigma^2}) = 1/2$ and accordingly

$$\mathrm{median}(|X|) = \sqrt{2\sigma^2}\mathrm{erf}^{-1}(1/2) \tag{37}$$

by applying the inverse error function erf^{-1}. However, this can be simplified by deriving erf^{-1}. It can be shown that

$$\Phi(x) = \frac{1}{2}\left(1 + \mathrm{erf}\left(\frac{x}{\sqrt{2}}\right)\right)$$

with the probability distribution Φ of the standard normal distribution. This equation can be transformed to

$$\mathrm{erf}^{-1}(2\Phi(x) - 1) = \frac{x}{\sqrt{2}}.$$

Using the substitution $y = 2\Phi(x) - 1$ and accordingly $x = \Phi^{-1}((y + 1)/2)$, one obtains

$$\mathrm{erf}^{-1}(y) = \frac{\Phi^{-1}\left(\frac{y+1}{2}\right)}{\sqrt{2}}.$$

It follows that $\mathrm{erf}^{-1}(1/2) = z_{3/4}/\sqrt{2}$ where $z_{3/4}$ is the third quartile of the standard normal distribution. Finally, for the median of $|X|$ as presented in Eq. (37) it applies that

$$\mathrm{median}(|X|) = z_{3/4}\sigma \approx 0.674\sigma.$$

In our case, X corresponds to T as presented in Eq. (33) and $\sigma = \sqrt{\lambda}$. Given a sample of n realisations $\hat{T}_1, \hat{T}_2, \ldots, \hat{T}_n$ of T under the null hypothesis, a robust estimator of λ according to [7] is

$$\left(\frac{\mathrm{median}(|\hat{T}_1|, |\hat{T}_2| \ldots, |\hat{T}_n|)}{0.675}\right)^2$$

or related [8]

$$\hat{\lambda} = \frac{\mathrm{median}(\hat{T}_1^2, \hat{T}_2^2 \ldots, \hat{T}_n^2)}{0.456}.$$

Similar to correcting the variance inflation of a squared test statistic T^2 by division of $\hat{\lambda}$ [7], it is possible to correct T as follows

$$T_{\mathrm{gc}} = \frac{T}{\sqrt{\hat{\lambda}}}.$$

After correction of the variance inflation of T distributed as presented in Eq. (33) under the null hypothesis, the test statistic T_{gc} is approximately standard normal

$$T_{\mathrm{gc}} \sim \mathrm{N}(0, 1)$$

and maintains the correct type I error of the test. In contrast, the distribution of T assuming the alternative hypothesis is given by Eq. (36). Correction by $\sqrt{\hat{\lambda}}$ yields

$$T_{\mathrm{gc}} \sim \mathrm{N}\left(\frac{\mu}{\sqrt{\hat{\lambda}}}, 1\right).$$

Inflation correction affects the expectation μ as given in Eq. (35) of the test statistic and reduces the power of the test unless λ is close to 1.

7 List of symbols

Symbol	Description
$\mathbf{0}$	vector of zeros
$\mathbf{b}$	vector comprising intercept and slope
b_1	intercept
b_2	slope
$\boldsymbol{\beta}$	vector comprising intercept and slope of the simplified model
β_1	intercept of the simplified model
β_2	slope of the simplified model
$\hat{\boldsymbol{\beta}}$	vector of beta estimates of the simplified model
$\hat{\beta}_2$	estimate of the slope of the simplified model
Cov	covariance
c	number of children
δ	Kronecker delta
δ	probability that both alleles are inherited from a common ancestor
E	expected value
$\mathbf{e}$	vector of residuals
erf	error function
$\boldsymbol{\epsilon}$	vector of residuals of the simplified model
$\mathbf{F}$	covariance matrix of $\mathbf{f}$
F_X	cumulative distribution function of X
$\mathbf{f}$	vector of sums of polygenic effects and residuals
f	number of families/fathers
f_X	probability density function of X
Φ	cumulative distribution function of the standard normal distribution
ϕ	probability that only one allele is inherited from a common ancestor
$\mathbf{G}$	relatedness matrix
$\mathbf{g}$	vector of polygenic effects
G_2	sum of squared elements
G_r	sum of squared row sums
G_s	function of G_2, G_r and n
$\bar{G}$	mean relatedness
$\mathbf{I}$	identity matrix
λ	variance inflation factor
λ'	expected λ
$\lambda'_{f;m;c}$	expected λ for a family study with f fathers, m mothers and c children
λ'_t	expected variance inflation obtained by transformation
$\hat{\lambda}$	estimate of λ obtained from a sample of T statistics
$\mathbf{M}$	idempotent matrix
m	number of mothers
μ	expected value of the normal distribution

Symbol	Description
N	normal distribution
n	number of samples
ν	deflation factor of the empirical variance estimate of beta
p	allele frequency of the reference allele
q	allele frequency of the respective other allele
R_h^2	heritability
R_s^2	explained variance by the SNP
R_t^2	heritability obtained by transformation
$\mathbf{S}$	matrix with a column vector equal to **1** and a vector with SNP genotypes
S_s^2	empirical variance of the SNP genotypes
$S_\upbeta^2$	empirical variance of the beta estimate of the simplified model
S_ϵ^2	empirical residual variance of the simplified model
$\mathbf{s}$	vector of SNP genotypes
s_q	sum of squared differences from the mean
$\bar{s}$	mean
$\tilde{s}$	mean of squared elements
σ^2	variance of a normal distribution
σ_e^2	variance of residuals
σ_ϵ^2	variance of residuals of the simplified model
σ_g^2	variance of polygenic effects
T	matrix transposition operator when used as exponent
T	T statistic
T_gc	T statistic corrected by genomic control
$\hat{T}$	realisation of T
tr	matrix trace
V	variance
$V_\upbeta$	variance of the beta estimate without heritability
X	random variable
$\mathbf{y}$	vector of observed phenotypes
$z_{3/4}$	third quartile of the standard normal distribution

References

[1] Aulchenko, Y.S., de Koning, D.J., Haley, C.: Genomewide rapid association using mixed model and regression: a fast and simple method for genomewide pedigree-based quantitative trait loci association analysis. Genetics **177**(1), 577–585 (2007). doi:10.1534/genetics.107.075614

[2] Boerwinkle, E., Chakraborty, R., Sing, C.F.: The use of measured genotype information in the analysis of quantitative phenotypes in man. I. Models and analytical methods. Ann. Hum. Genet. **50**(Pt 2), 181–194 (1986). doi:10.1111/j.1469-1809.1986.tb01037.x

[3] Wang, J.: An estimator for pairwise relatedness using molecular markers. Genetics **160**(3), 1203–1215 (2002)

[4] Stuart, A., Ord, K., Arnold, S.: Kendall's Advanced Theory of Statistics vol. 2A, 6th edn. Arnold, a member of the Hodder Headline Group, 338 Euston Road, London NW1 3BH (1999)

[5] Maxima: a Computer Algebra System. Version 5.38.1. `http://maxima.sourceforge.net/`. Accessed 14 Mar 2017.

[6] Czado, C., Schmidt, T.: Mathematische Statistik. Statistik und ihre Anwendungen. Springer, Heidelberg (2011). doi:10.1007/978-3-642-17261-8

[7] Devlin, B., Roeder, K.: Genomic control for association studies. Biometrics **55**(4), 997–1004 (1999). doi:10.1111/j.0006-341X.1999.00997.x

[8] Amin, N., van Duijn, C.M., Aulchenko, Y.S.: A genomic background based method for association analysis in related individuals. PLoS ONE **2**(12), 1274 (2007). doi:10.1371/journal.pone.0001274
