# Supplementary material for: On the impact of relatedness on SNP association analysis
Source: BMC Genet. 2017 Dec 6;18:104. doi: 10.1186/s12863-017-0571-x (PMC5719591; doi:10.1186/s12863-017-0571-x)
Supplement: Supplementary file 4 — Preparation of HapMap data. This document provides details regarding the filtering of samples and SNPs of the HapMap data. (PDF 97 kb) [file 12863_2017_571_MOESM4_ESM.pdf]

Preparation of the HapMap genotype data

July 11, 2017

We obtained the merged phase I+II+III release #28 (NCBI build 36, dbSNP b126) HapMap CEU genotype data consisting of 174 samples and 3,908,761 autosomal SNPs. HapMap genotype files for CEU samples and autosomal SNPs were downloaded from the HapMap data archive [1]. The HapMap project website is not longer available, however, genotype data can still be retrieved from the FTP server.

In a preliminary analysis we checked family assignment as provided by HapMap by estimating pairwise relatedness with the method presented in [2]. For analysis, we excluded 209 SNPs known as indels and 31 SNPs which map to multiple positions as suggested by the HapMap readme file. Additionally, we excluded 1,158,824 monomorphic SNPs. Since some of the SNPs fulfilled more than one exclusion criterion, we excluded 1,159,062 SNPs. Based on the remaining 2,749,699 autosomal SNPs, we attempted to estimate the pairwise relatedness of all 174 HapMap CEU samples. The matrix of pairwise relatedness estimates is provided as Additional File 5. We discovered three cluster of individuals: 81 individuals sharing common SNPs with all other individuals and clusters of 9 and 84 individuals which do not share any SNP between the clusters. This means that pairwise relatedness could not be checked for individuals between the latter two clusters. We are focusing on complete and confirmed trio data, therefore we excluded 24 individuals who belong to 8 trios comprising the 9 individuals of the smaller cluster. Further, we identified a first degree relationship between NA07045 belonging to trio NA06986, NA06997, NA07045 and NA12813 belonging to trio NA12801, NA12812, NA12813. This was also noted in [3]. We excluded all 3 individuals of trio NA06986, NA06997, NA07045 because of slightly more heterozygosity as compared to the other family. Finally, we excluded 18 individuals which are not members of (complete) trio families. The remaining 129 individuals belonging to 43 trios were used for analysis. Additional File 6 contains a detailed list of samples and the reason for exclusion where applicable.

Based on 129 individuals (43 trios) included in our study, filtering of 3,908,761 autosomal SNPs was done as follows. We excluded 209 SNPs known as indels and 31 SNPs which map to multiple positions as mentioned above. As we are focusing on a reliable set of SNPs measured in all individuals, i.e. 100% call rate, we excluded 2,888,347 SNPs not covering all samples. We checked for deviation from Hardy-Weinberg equilibrium by applying an exact test [4] on the 86 founder of the 43 trios and excluded one SNP with $p < 10^{-6}$. Since some of the SNPs fulfilled more than one exclusion criterion, 2,888,546

SNPs were excluded. Finally, 1,020,215 SNPs measured in 129 HapMap samples belonging to 43 trios were available. Additional File 7 provides a list of corresponding SNP identifiers.

References

[1] HapMap: Merged phase I+II and III genotype files. `ftp://ftp.ncbi.nlm.nih.gov/hapmap/genotypes/2010-08_phaseII+III/`. Accessed 14 Mar 2017.

[2] Wang, J.: An estimator for pairwise relatedness using molecular markers. Genetics **160**(3), 1203–1215 (2002)

[3] Pemberton, T.J., Wang, C., Li, J.Z., Rosenberg, N.A.: Inference of unexpected genetic relatedness among individuals in HapMap Phase III. Am. J. Hum. Genet. **87**(4), 457–464 (2010). doi:10.1016/j.ajhg.2010.08.014

[4] Louis, E.J., Dempster, E.R.: An exact test for Hardy-Weinberg and multiple alleles. Biometrics **43**(4), 805–811 (1987). doi:10.2307/2531534
