# Supplementary material for: On the impact of relatedness on SNP association analysis
Source: BMC Genet. 2017 Dec 6;18:104. doi: 10.1186/s12863-017-0571-x (PMC5719591; doi:10.1186/s12863-017-0571-x)
Supplement: Supplementary file 9 — Comparison of different degrees of heritability. This file contains additional tables with inflation results for different degrees of heritability. (PDF 75 kb) [file 12863_2017_571_MOESM9_ESM.pdf]

Comparison of different degrees of heritability

October 20, 2017

R^2_{h}	Study	n	$\bar{\lambda}$	$\bar{\lambda}_{10\%}$	λ'	$\lambda'_{f;m;c}$	$\bar{G}$	R^2_{t}
0.3	HapMap	129	1.096 (0.025)	1.098 (0.017)	1.099	-	0.006	0.152
0.6	HapMap	129	1.192 (0.049)	1.197 (0.034)	1.198	-	0.006	0.152
0.9	HapMap	129	1.288 (0.074)	1.295 (0.051)	1.297	-	0.006	0.152
0.3	SFS1	129	1.095 (0.029)	1.098 (0.017)	1.098	1.098	0.007	0.153
0.6	SFS1	129	1.189 (0.058)	1.195 (0.034)	1.196	1.197	0.007	0.153
0.9	SFS1	129	1.284 (0.087)	1.293 (0.051)	1.294	1.295	0.007	0.153
0.3	SFS2	999	1.102 (0.017)	1.104 (0.007)	1.105	1.100	0.001	0.143
0.6	SFS2	999	1.204 (0.033)	1.209 (0.013)	1.209	1.200	0.001	0.143
0.9	SFS2	999	1.306 (0.050)	1.313 (0.020)	1.314	1.299	0.001	0.143
0.3	Sorbs	977	1.137 (0.045)	1.149 (0.024)	1.150	-	0.001	0.100
0.6	Sorbs	977	1.273 (0.090)	1.298 (0.047)	1.299	-	0.001	0.100
0.9	Sorbs	977	1.410 (0.135)	1.448 (0.071)	1.449	-	0.001	0.100
0.3	SFS3	999	1.335 (0.046)	1.341 (0.028)	1.340	1.334	0.002	0.044
0.6	SFS3	999	1.671 (0.093)	1.681 (0.055)	1.681	1.668	0.002	0.044
0.9	SFS3	999	2.006 (0.139)	2.022 (0.083)	2.021	2.002	0.002	0.044

Table 1: Estimated variance inflation under relatedness for different degrees of heritability R^2_{h}. Variance inflation and related measures are compared between the data sets HapMap, SFS1 (synthetic family study 1), SFS2, Sorbs and SFS3. Provided are the sample size n, average inflation $\bar{\lambda}$ of all SNPs, average inflation $\bar{\lambda}_{10\%}$ estimated for SNPs with minor allele frequencies $>$ 10%, expected (theoretical) inflation λ' obtained from estimated relationships, expected inflation $\lambda'_{f;m;c}$ obtained from true relationships (synthetic family studies only), mean relatedness $\bar{G}$ and heritability R^2_{t} corresponding to inflation $\lambda'_{\mathrm{t}} = 1.05$. Standard deviations are given in parentheses.

R_h^2	Study	$\bar{T}$	$\bar{S}^2$	ν
0.3	HapMap	0.002 (0.034)	1.122 (0.058)	0.997
0.6	HapMap	0.002 (0.035)	1.225 (0.075)	0.995
0.9	HapMap	0.002 (0.037)	1.330 (0.096)	0.992
0.3	SFS1	-0.000 (0.033)	1.115 (0.058)	0.997
0.6	SFS1	-0.000 (0.035)	1.217 (0.080)	0.994
0.9	SFS1	-0.000 (0.037)	1.321 (0.107)	0.992
0.3	SFS2	-0.001 (0.034)	1.103 (0.053)	1.000
0.6	SFS2	-0.001 (0.035)	1.206 (0.063)	0.999
0.9	SFS2	-0.001 (0.037)	1.309 (0.076)	0.999
0.3	Sorbs	-0.001 (0.033)	1.138 (0.066)	1.000
0.6	Sorbs	-0.001 (0.035)	1.275 (0.103)	0.999
0.9	Sorbs	-0.001 (0.037)	1.412 (0.144)	0.999
0.3	SFS3	0.001 (0.036)	1.339 (0.076)	0.999
0.6	SFS3	0.001 (0.040)	1.677 (0.119)	0.998
0.9	SFS3	0.001 (0.043)	2.015 (0.166)	0.997

Table 2: Simulation results for the test statistic T under the null hypothesis for different degrees of heritability R_h^2. The test statistics $\bar{T}$ averaged over replicates and SNPs and the average of the empirical variances $\bar{S}^2$ are compared between HapMap, SFS1 (synthetic family study 1), SFS2, Sorbs and SFS3 assuming the null hypothesis. Standard deviations are presented in parentheses. We further provide an estimate of the deflation factor ν for the empirical variance of the beta estimate.

R_h^2	Study	$\bar{T}$	$\bar{S}^2$	μ
0.3	HapMap	1.613 (0.034)	1.133 (0.057)	1.600
0.6	HapMap	1.616 (0.035)	1.237 (0.074)	1.600
0.9	HapMap	1.619 (0.037)	1.343 (0.095)	1.600
0.3	SFS1	1.612 (0.033)	1.127 (0.061)	1.600
0.6	SFS1	1.615 (0.035)	1.230 (0.085)	1.600
0.9	SFS1	1.619 (0.036)	1.336 (0.112)	1.600
0.3	SFS2	4.472 (0.033)	1.121 (0.052)	4.468
0.6	SFS2	4.472 (0.035)	1.225 (0.063)	4.468
0.9	SFS2	4.472 (0.036)	1.330 (0.076)	4.468
0.3	Sorbs	4.423 (0.035)	1.152 (0.068)	4.418
0.6	Sorbs	4.421 (0.037)	1.292 (0.106)	4.418
0.9	Sorbs	4.420 (0.039)	1.432 (0.148)	4.418
0.3	SFS3	4.474 (0.038)	1.347 (0.075)	4.468
0.6	SFS3	4.477 (0.042)	1.687 (0.117)	4.468
0.9	SFS3	4.479 (0.046)	2.030 (0.162)	4.468

Table 3: Simulation results for the test statistic T under the alternative hypothesis for different degrees of heritability R_h^2. The test statistics $\bar{T}$ averaged over replicates and SNPs and the average of the empirical variances $\bar{S}^2$ are compared between HapMap, SFS1 (synthetic family study 1), SFS2, Sorbs and SFS3 assuming the alternative hypothesis with $R_\mathrm{s}^2 = 0.02$. Standard deviations are presented in parentheses. We further provide the expected value μ of the test statistic T.
