# Supplementary material for: On the impact of relatedness on SNP association analysis
Source: BMC Genet. 2017 Dec 6;18:104. doi: 10.1186/s12863-017-0571-x (PMC5719591; doi:10.1186/s12863-017-0571-x)
Supplement: Supplementary file 10 — Comparison of methods for modelling the polygenic effect. This file provides additional tables with inflation results for different polygenic models. (PDF 67 kb) [file 12863_2017_571_MOESM10_ESM.pdf]

Comparison of methods for modelling the polygenic effect

October 20, 2017

We compare two methods for modelling the polygenic effect. In the manuscript, we focussed on modelling the polygenic effect by a multivariate normal distribution “mvn”. Alternatively, single SNPs could be used to model the polygenic effect. For this purpose, we assumed m random SNPs ($m \in \{1, 2, 3\}$), each explaining a fraction R^2_{h}/m of the variance of the phenotype. Results of test statistics under the null and alternative are shown below and are very similar between the scenarios.

Polygenic model	Study	$\bar{T}$	$\bar{S}^2$	ν
1	HapMap	-0.001 (0.037)	1.337 (0.150)	0.992
2	HapMap	0.002 (0.038)	1.320 (0.095)	0.992
3	HapMap	-0.001 (0.037)	1.320 (0.092)	0.992
mvn	HapMap	0.002 (0.037)	1.330 (0.096)	0.992
1	SFS1	-0.001 (0.038)	1.343 (0.218)	0.992
2	SFS1	0.001 (0.038)	1.320 (0.111)	0.992
3	SFS1	-0.002 (0.036)	1.318 (0.108)	0.992
mvn	SFS1	-0.000 (0.037)	1.321 (0.107)	0.992
1	SFS2	-0.000 (0.038)	1.310 (0.297)	0.999
2	SFS2	-0.000 (0.036)	1.302 (0.078)	0.999
3	SFS2	-0.002 (0.037)	1.298 (0.078)	0.999
mvn	SFS2	-0.001 (0.037)	1.309 (0.076)	0.999
1	Sorbs	0.001 (0.038)	1.459 (0.830)	0.999
2	Sorbs	-0.001 (0.040)	1.406 (0.214)	0.999
3	Sorbs	-0.001 (0.039)	1.412 (0.192)	0.999
mvn	Sorbs	-0.001 (0.037)	1.412 (0.144)	0.999
1	SFS3	-0.001 (0.042)	1.997 (0.173)	0.997
2	SFS3	0.002 (0.046)	1.999 (0.166)	0.997
3	SFS3	-0.001 (0.044)	2.001 (0.156)	0.997
mvn	SFS3	0.001 (0.043)	2.015 (0.166)	0.997

Table 1: Simulation results for the test statistic T under the null hypothesis for different polygenic models. The test statistics $\bar{T}$ averaged over replicates and SNPs and the average of the empirical variances $\bar{S}^2$ are compared between HapMap, SFS1 (synthetic family study 1), SFS2, Sorbs and SFS3 assuming the null hypothesis and $R^2_{\text{h}} = 0.9$. Standard deviations are presented in parentheses. We further provide an estimate of the deflation factor ν for the empirical variance of the beta estimate.

Polygenic model	Study	$\bar{T}$	$\bar{S}^2$	μ
1	HapMap	1.605 (0.036)	1.334 (0.148)	1.600
2	HapMap	1.609 (0.038)	1.324 (0.095)	1.600
3	HapMap	1.611 (0.035)	1.329 (0.089)	1.600
mvn	HapMap	1.619 (0.037)	1.343 (0.095)	1.600
1	SFS1	1.604 (0.036)	1.352 (0.244)	1.600
2	SFS1	1.610 (0.036)	1.324 (0.109)	1.600
3	SFS1	1.613 (0.036)	1.327 (0.107)	1.600
mvn	SFS1	1.619 (0.036)	1.336 (0.112)	1.600
1	SFS2	4.468 (0.035)	1.302 (0.085)	4.468
2	SFS2	4.470 (0.036)	1.308 (0.077)	4.468
3	SFS2	4.471 (0.037)	1.311 (0.080)	4.468
mvn	SFS2	4.472 (0.036)	1.330 (0.076)	4.468
1	Sorbs	4.421 (0.040)	1.453 (0.704)	4.418
2	Sorbs	4.421 (0.038)	1.412 (0.187)	4.418
3	Sorbs	4.424 (0.036)	1.420 (0.174)	4.418
mvn	Sorbs	4.420 (0.039)	1.432 (0.148)	4.418
1	SFS3	4.471 (0.046)	1.998 (0.163)	4.468
2	SFS3	4.472 (0.046)	2.005 (0.165)	4.468
3	SFS3	4.475 (0.045)	2.009 (0.157)	4.468
mvn	SFS3	4.479 (0.046)	2.030 (0.162)	4.468

Table 2: Simulation results for the test statistic T under the alternative hypothesis for different polygenic models. The test statistics $\bar{T}$ averaged over replicates and SNPs and the average of the empirical variances $\bar{S}^2$ are compared between HapMap, SFS1 (synthetic family study 1), SFS2, Sorbs and SFS3 assuming the alternative hypothesis with $R_s^2 = 0.02$ and heritability $R_h^2 = 0.9$. Standard deviations are presented in parentheses. We further provide the expected value μ of the test statistic T.
