# Supplementary material for: On the impact of relatedness on SNP association analysis
Source: BMC Genet. 2017 Dec 6;18:104. doi: 10.1186/s12863-017-0571-x (PMC5719591; doi:10.1186/s12863-017-0571-x)
Supplement: Supplementary file 11 — Comparison of different relatedness estimators. This document summarizes different methods for estimating relatedness, presents corresponding inflation results and shows the impact of small allele frequencies on relatedness estimates. (PDF 140 kb) [file 12863_2017_571_MOESM11_ESM.pdf]

Comparison of different relatedness estimators

October 20, 2017

Here, we study the impact of three different methods for estimation of pairwise relatedness on our results. The method denoted as "Wang" refers to the estimator we applied in our main analysis. For the full theory, see [1]. Relatedness can also be calculated by twice the kinship matrix as estimated via Equation 2.2 in [2]. Finally, relatedness assessed by IBS (identical by state) of alleles from a pair of individuals is estimated as follows: The pairwise IBS matrix $\mathbf{M}$ is estimated via Equation 2.3 in [2] and relatedness is calculated by $2(\mathbf{M} - \bar{M})/(1 - \bar{M})$, where $\bar{M}$ corresponds to the average IBS of two individuals from the same sample. See also [3, 4]. For the null and the alternative hypothesis, it revealed that the estimating method of the relatedness matrix has a negligible impact on the inflation results as shown in Tables 1-3 below.

Estimator	Study	n	$\bar{\lambda}$	$\bar{\lambda}_{10\%}$	λ'	$\lambda'_{f;m;c}$	$\bar{G}$	R^2_{t}
IBS	HapMap	129	1.295 (0.074)	1.302 (0.051)	1.303	-	0.000	0.149
kinship	HapMap	129	1.297 (0.073)	1.303 (0.051)	1.285	-	-0.008	0.158
Wang	HapMap	129	1.288 (0.074)	1.295 (0.051)	1.297	-	0.006	0.152
IBS	SFS1	129	1.291 (0.087)	1.300 (0.051)	1.301	1.295	-0.000	0.149
kinship	SFS1	129	1.291 (0.085)	1.300 (0.050)	1.280	1.295	-0.008	0.161
Wang	SFS1	129	1.284 (0.087)	1.293 (0.051)	1.294	1.295	0.007	0.153
IBS	SFS2	999	1.306 (0.050)	1.313 (0.020)	1.314	1.299	0.000	0.144
kinship	SFS2	999	1.304 (0.049)	1.307 (0.019)	1.303	1.299	-0.001	0.149
Wang	SFS2	999	1.306 (0.050)	1.313 (0.020)	1.314	1.299	0.001	0.143
IBS	Sorbs	977	1.412 (0.131)	1.448 (0.071)	1.448	-	0.000	0.100
kinship	Sorbs	977	1.383 (0.120)	1.412 (0.063)	1.382	-	-0.001	0.118
Wang	Sorbs	977	1.410 (0.135)	1.448 (0.071)	1.449	-	0.001	0.100
IBS	SFS3	999	2.008 (0.139)	2.023 (0.083)	2.024	2.002	-0.000	0.044
kinship	SFS3	999	1.998 (0.136)	2.009 (0.081)	1.997	2.002	-0.001	0.045
Wang	SFS3	999	2.006 (0.139)	2.022 (0.083)	2.021	2.002	0.002	0.044

Table 1: Estimated variance inflation under relatedness for different relatedness estimators. Variance inflation and related measures are compared between the data sets HapMap, SFS1 (synthetic family study 1), SFS2, Sorbs and SFS3 assuming $R^2_{\mathrm{h}} = 0.9$. Provided are the sample size n, average inflation $\bar{\lambda}$ of all SNPs, average inflation $\bar{\lambda}_{10\%}$ estimated for SNPs with minor allele frequencies $> 10\%$, expected (theoretical) inflation λ' obtained from estimated relationships, expected inflation $\lambda'_{f;m;c}$ obtained from true relationships (synthetic family studies only), mean relatedness $\bar{G}$ and heritability R^2_{t} corresponding to inflation $\lambda'_{\mathrm{t}} = 1.05$. Standard deviations are given in parentheses.

Estimator	Study	$\bar{T}$	$\bar{S}^2$	ν
IBS	HapMap	0.002 (0.037)	1.329 (0.096)	0.998
kinship	HapMap	0.002 (0.037)	1.322 (0.094)	1.005
Wang	HapMap	0.002 (0.037)	1.330 (0.096)	0.992
IBS	SFS1	-0.000 (0.037)	1.320 (0.107)	0.998
kinship	SFS1	-0.000 (0.036)	1.311 (0.104)	1.005
Wang	SFS1	-0.000 (0.037)	1.321 (0.107)	0.992
IBS	SFS2	-0.001 (0.037)	1.309 (0.076)	1.000
kinship	SFS2	-0.001 (0.037)	1.306 (0.075)	1.001
Wang	SFS2	-0.001 (0.037)	1.309 (0.076)	0.999
IBS	Sorbs	-0.001 (0.037)	1.419 (0.137)	1.000
kinship	Sorbs	-0.001 (0.037)	1.383 (0.132)	1.001
Wang	Sorbs	-0.001 (0.037)	1.412 (0.144)	0.999
IBS	SFS3	0.001 (0.043)	2.014 (0.165)	0.999
kinship	SFS3	0.001 (0.043)	2.003 (0.162)	1.000
Wang	SFS3	0.001 (0.043)	2.015 (0.166)	0.997

Table 2: Simulation results for the test statistic T under the null hypothesis for different relatedness estimators. The test statistics $\bar{T}$ averaged over replicates and SNPs and the average of the empirical variances $\bar{S}^2$ are compared between HapMap, SFS1 (synthetic family study 1), SFS2, Sorbs and SFS3 assuming the null hypothesis and $R_{\mathrm{h}}^2 = 0.9$. Standard deviations are presented in parentheses. We further provide an estimate of the deflation factor ν for the empirical variance of the beta estimate.

Estimator	Study	$\bar{T}$	$\bar{S}^2$	μ
IBS	HapMap	1.615 (0.036)	1.342 (0.095)	1.600
kinship	HapMap	1.609 (0.036)	1.334 (0.094)	1.600
Wang	HapMap	1.619 (0.037)	1.343 (0.095)	1.600
IBS	SFS1	1.614 (0.036)	1.335 (0.112)	1.600
kinship	SFS1	1.608 (0.036)	1.325 (0.110)	1.600
Wang	SFS1	1.619 (0.036)	1.336 (0.112)	1.600
IBS	SFS2	4.470 (0.036)	1.330 (0.076)	4.468
kinship	SFS2	4.471 (0.036)	1.327 (0.075)	4.468
Wang	SFS2	4.472 (0.036)	1.330 (0.076)	4.468
IBS	Sorbs	4.412 (0.039)	1.439 (0.141)	4.418
kinship	Sorbs	4.423 (0.039)	1.402 (0.136)	4.418
Wang	Sorbs	4.420 (0.039)	1.432 (0.148)	4.418
IBS	SFS3	4.474 (0.046)	2.029 (0.162)	4.468
kinship	SFS3	4.475 (0.046)	2.018 (0.158)	4.468
Wang	SFS3	4.479 (0.046)	2.030 (0.162)	4.468

Table 3: Simulation results for the test statistic T under the alternative hypothesis for different relatedness estimators. The test statistics $\bar{T}$ averaged over replicates and SNPs and the average of the empirical variances $\bar{S}^2$ are compared between HapMap, SFS1 (synthetic family study 1), SFS2, Sorbs and SFS3 assuming the alternative hypothesis with $R_{\mathrm{s}}^2 = 0.02$ and heritability $R_{\mathrm{h}}^2 = 0.9$. Standard deviations are presented in parentheses. We further provide the expected value μ of the test statistic T.

We also compared the components of the relatedness matrices between the methods (see Figure 1). Comparison is based on 977 Sorbs and the same 100,000 SNPs as used in our main analysis. For assessing the impact of small allele frequencies, we re-estimated pairwise relatedness based on the subset of 71,705 SNPs with minor allele frequencies (maf) $> 10\%$. Although the overall agreement of the estimators is good, it further improves for variants with maf $> 10\%$. This can be traced back to higher robustness of the Wang estimator regarding low frequency variants. Indeed, this estimator was intended to be more robust, and therefore, was used in the main analysis.

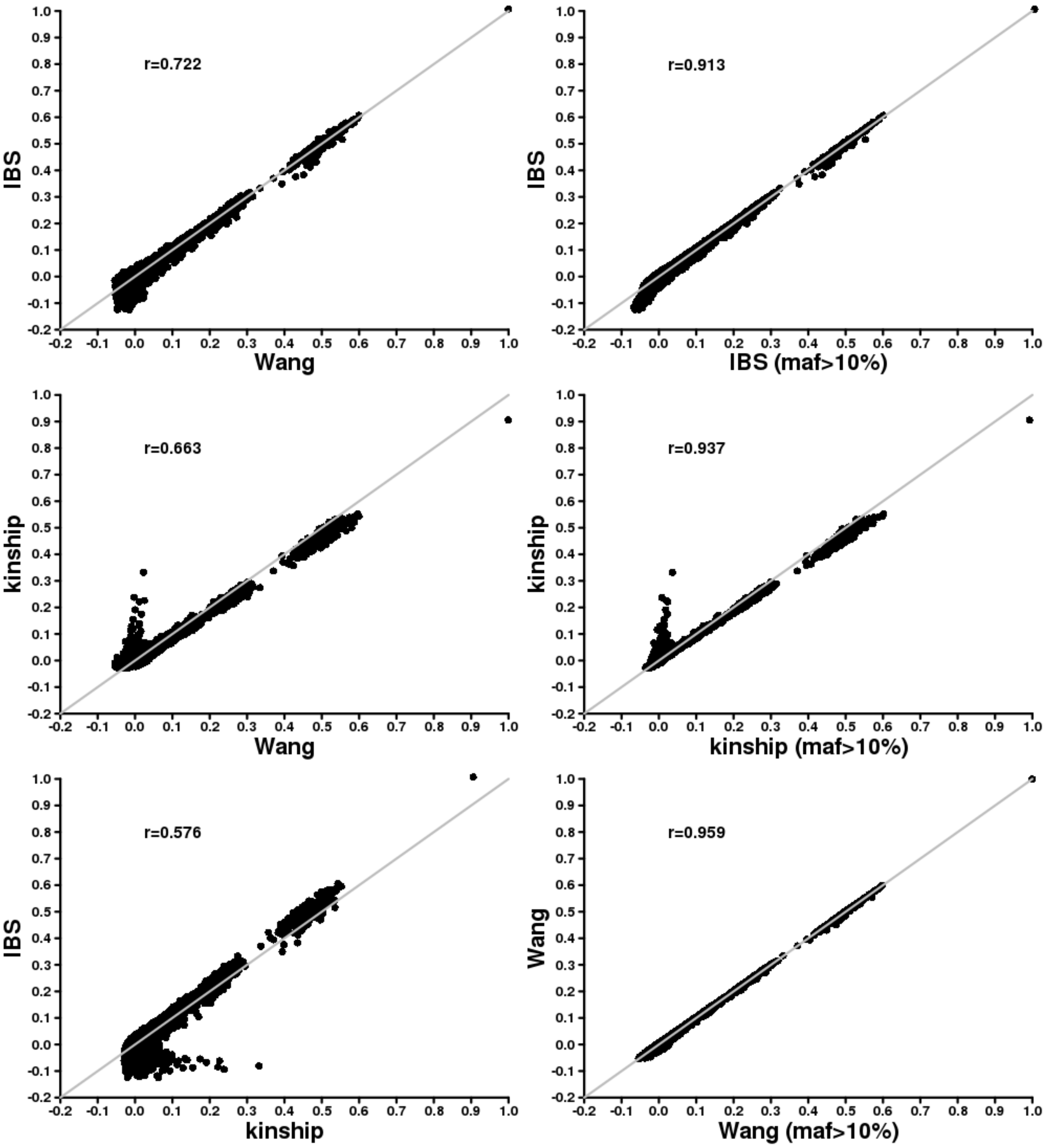

Figure 1: Pairwise comparison of estimators. On the left different estimators are compared, whereas on the right estimates of the same estimator are compared. The Spearman correlation is shown for each comparison.

References

[1] Wang, J.: An estimator for pairwise relatedness using molecular markers. Genetics **160**(3), 1203–1215 (2002)

[2] Astle, W., Balding, D.J.: Population Structure and Cryptic Relatedness in Genetic Association Studies. Statist. Sci. **24**(4), 451–471 (2009). doi:10.1214/09-STS307

[3] Yu, J., Pressoir, G., Briggs, W.H., Vroh Bi, I., Yamasaki, M., Doebley, J.F., McMullen, M.D., Gaut, B.S., Nielsen, D.M., Holland, J.B., Kresovich, S., Buckler, E.S.: A unified mixed-model method for association mapping that accounts for multiple levels of relatedness. Nat. Genet. **38**(2), 203–208 (2006). doi:10.1038/ng1702

[4] Zhao, K., Aranzana, M.J., Kim, S., Lister, C., Shindo, C., Tang, C., Toomajian, C., Zheng, H., Dean, C., Marjoram, P., Nordborg, M.: An Arabidopsis example of association mapping in structured samples. PLoS Genet. **3**(1), 4 (2007). doi:10.1371/journal.pgen.0030004
